# Habitat degradation and indiscriminate hunting differentially impact faunal communities in the Southeast Asian tropical biodiversity hotspot

**DOI:** 10.1038/s42003-019-0640-y

**Published:** 2019-10-30

**Authors:** Andrew Tilker, Jesse F. Abrams, Azlan Mohamed, An Nguyen, Seth T. Wong, Rahel Sollmann, Jürgen Niedballa, Tejas Bhagwat, Thomas N. E. Gray, Benjamin M. Rawson, Francois Guegan, Johnny Kissing, Martin Wegmann, Andreas Wilting

**Affiliations:** 1 Department of Ecological Dynamics, Leibniz Institute for Zoo and Wildlife Research, Berlin, 10315 Germany; 2 Global Wildlife Conservation, Austin, TX 78767 USA; 3 World Wide Fund for Nature, 46150 Petaling Jaya, Selangor Malaysia; 4 Department of Wildlife, Fish, and Conservation Biology, University of California Davis, Davis, CA 95616 USA; 5 Wildlife Alliance, 10018 Broadway, NY USA; 6 World Wide Fund for Nature, Hanoi, Vietnam; 7 World Wide Fund for Nature, Vientiane, Lao PDR; 8 Sabah Forestry Department, Sandakan, 90009 Sabah Malaysia; 9 Department of Remote Sensing, University of Würzburg, 97074 Würzburg, Germany

**Keywords:** Tropical ecology, Biodiversity, Conservation biology

## Abstract

Habitat degradation and hunting have caused the widespread loss of larger vertebrate species (defaunation) from tropical biodiversity hotspots. However, these defaunation drivers impact vertebrate biodiversity in different ways and, therefore, require different conservation interventions. We conducted landscape-scale camera-trap surveys across six study sites in Southeast Asia to assess how moderate degradation and intensive, indiscriminate hunting differentially impact tropical terrestrial mammals and birds. We found that functional extinction rates were higher in hunted compared to degraded sites. Species found in both sites had lower occupancies in the hunted sites. Canopy closure was the main predictor of occurrence in the degraded sites, while village density primarily influenced occurrence in the hunted sites. Our findings suggest that intensive, indiscriminate hunting may be a more immediate threat than moderate habitat degradation for tropical faunal communities, and that conservation stakeholders should focus as much on overhunting as on habitat conservation to address the defaunation crisis.

## Abstract (web summary)

Andrew Tilker, Jesse F. Abrams et al. assessed habitat degradation and hunting on tropical terrestrial mammals and birds in Southeast Asia. They find higher levels of functional extinction and consistently lower animal occupancy in hunted sites, suggesting that indiscriminate hunting is a more immediate threat for tropical animal communities.

## Introduction

Global biodiversity is decreasing at an alarming rate^1^, with the most rapid declines occurring in tropical rainforests^2^. Two overarching threats, habitat alteration^3^ and hunting^4^, have led to the widespread loss of larger vertebrate species (defaunation) from tropical biodiversity hotspots. Both defaunation drivers result in impoverished and homogenized faunal communities, with myriad ecological, evolutionary, and socio-economic consequences^5^. Defaunation drives population declines^6^ and species extinctions^7^, alters community- and ecosystem-level processes^8^, changes the trajectory of evolutionary pathways^9^, and threatens livelihoods for forest-dependent peoples^10^. There is mounting evidence in the scientific literature that, among global issues that have emerged from the Anthropocene, few have such diverse and potentially irremediable impacts^11^. To protect biodiversity and preserve ecosystem functions in the world’s remaining tropical rainforests, it is therefore imperative that conservation stakeholders devise effective solutions to address ever-increasing rates of habitat alteration and hunting.

Although both defaunation drivers cause species declines and alter mammal and bird communities, the mechanisms through which they operate are fundamentally different. Habitat alteration impacts ecological suitability by altering forest structure. Habitat alteration occurs along a gradient of degradation, ranging from forest conversion that results in the complete loss of suitable habitat^12^, to reduced impact selective logging that maintains overall forest structural integrity^13^. The extent of habitat loss in tropical rainforests through conversion^14^, and its effects on faunal communities^15^, have been well documented. Because complete habitat loss typically results in severe declines in vertebrate richness^16^, and has been linked to numerous local extinction events^17^, preventing deforestation has become a central theme of global conservation efforts focused on biodiversity protection in tropical rainforests. The impact of less extreme forms of habitat alteration on faunal communities is more complex, as different logging regimes result in varying levels of degradation. Several studies indicate that while levels of habitat degradation have a holistically negative impact on tropical mammal and bird communities, species-specific responses can vary substantially^18–20^. For example, forest specialists may decline with habitat degradation, leading to an overall decrease in species richness, even while some generalists benefit^21^. Even with several insightful studies on this topic in recent years, further research is needed to understand how mammal and bird communities respond to different levels of degradation and, more generally, the role of habitat degradation in pantropical faunal declines.

In addition to habitat degradation, there is increasing evidence that widespread and intensive hunting across the tropics has resulted in faunal declines^4^. However, the true extent of overhunting, and its specific impacts on faunal communities, remains poorly understood. This lack of information is partly due to the fact that hunting is linked to a diverse set of socio-economic and cultural drivers^22^, which tend to be manifested in regionally-specific patterns of wildlife exploitation. In this respect, hunting represents a more complex phenomenon than habitat degradation. Some patterns do however appear to be consistent across sites. Larger mammals appear to be particularly susceptible to overhunting, both because they are targeted by hunters^23^ and often have lower population densities^24^. There is also evidence that more eurytopic species show greater resilience to hunting pressure, as indicated by the survival of some generalist species in faunally impoverished systems^25^. Notably, much of the information in the scientific literature on the effects of hunting comes from sites where gun hunting is the predominant method of wildlife exploitation^26–28^. As a selective method, gun hunting is unlikely to directly impact entire faunal communities. The true consequences of more deleterious forms of hunting, such as indiscriminate snaring, have received far less attention and thus it remains largely unknown how overhunting by snaring impacts mammalian and ground-dwelling bird community structure and composition. Given that snaring levels are expected to increase in developing countries as regional bushmeat industries becomes increasingly commercialized^29^, further research into the impacts of nonselective hunting is needed.

Among the world’s tropical biodiversity hotspots, Southeast Asia is unique, both because of its exceptionally high levels of species richness and endemism, and the magnitude of the anthropogenic threats that it faces^30^. However, even within this hotspot, biodiversity and threat levels are not uniform. The island of Borneo and the Annamite Mountains of Vietnam and Laos stand out as sub-regional centers of endemism, especially for the region’s mammals and birds^31^. At least three small carnivores, one muntjac, and five galliforms are found only on Borneo^32,33^. The Annamites ecoregion contains similarly high concentrations of endemic mammals and birds^34^. Remarkably, several species restricted to this ecoregion were only recently discovered by science, including the saola *Pseudoryx nghetinhensis*^35^, the large-antlered muntjac *Muntiacus vuquangensis*^36^, and the Annamite striped rabbit *Nesolagus timminsi*^37,38^. The two regions face significant, although fundamentally different, anthropogenic pressures. The primary threat to faunal communities in many parts of Borneo is widespread habitat alteration. Over the past forty years, Borneo’s forests have had one of the highest rates of commercial logging of any tropical region in the world, with much of its remaining rainforests degraded^39^. Although hunting is an issue in certain parts of Borneo^40^, all available evidence indicates that levels of hunting pressure in most parts of Borneo are significantly lower than the levels of industrial-scale exploitation found in mainland Southeast Asia. In contrast, hunting pressure is extremely high in the Annamites, where intensive hunting is predominantly accomplished by the setting of indiscriminate wire snares^41^. Snaring is almost ubiquitous across Annamites forests, even in protected areas^41^, and has led to precipitous declines in the populations of the region’s terrestrial mammals and birds.

Understanding the impacts of indiscriminate hunting and habitat degradation on tropical mammal and bird communities is essential to the development of effective mitigation strategies. Knowledge on the effects of specific defaunation drivers allows conservation stakeholders to make more informed management decisions, which can, in turn, optimize the efficacy of limited conservation resources. Although several studies have focused on the impacts of each driver, often focusing on one or two species of particular concern^42,43^, there have been no comprehensive, systematic, large-scale studies comparing how hunting and habitat degradation differentially impact tropical faunal communities. To address this question, we conducted landscape-scale systematic camera-trapping across six study sites in Southeast Asia that are characterized by different defaunation drivers. In Sabah, Malaysian Borneo, we surveyed three active or former logging concessions. The concessions have undergone varying levels of logging intensity, ranging from conventional logging to reduced impact sustainably-managed programs, resulting in a gradient of habitat degradation^44–46^. In contrast to most other areas in Southeast Asia, none of the areas has been subjected to significant past or current hunting pressure. In the Annamites, we surveyed two forest blocks (Bach Ma National Park [NP] and Hue/Quang Nam Saola Nature Reserves [SNRs]) in Vietnam and one block in Laos (consisting of Eastern Xe Sap National Protected [NPA] area and Palé watershed protection forest). Although these areas experienced extensive degradation during and shortly after the American-Vietnam war, habitat degradation over the last 30 years has been minimal^47^, and the areas are predominantly characterized by mature secondary forest. Unlike Malaysian Borneo, both past and current levels of hunting pressure are high, with most hunting accomplished by indiscriminate snaring^41,48,49^.

Here, we investigated how moderate habitat degradation and intensive, indiscriminate hunting differentially impact tropical faunal communities, with the ultimate goal of providing information that can support the development of more effective conservation strategies. We assessed defaunation in both hunted and degraded sites, and at three hierarchical levels: species’ functional extinction, species’ occurrence, and drivers of species’ occurrence. In all our hunted sites there is widespread, industrial snaring. Although our most degraded study site was subject to intensive conventional logging, altogether the degraded sites have experienced moderate levels of habitat disturbance, in the context that none were clear-cut. We used a defaunation index^50^ and Bayesian community occupancy models^51^ to evaluate defaunation at each level. Our setup, with three degraded but unhunted sites, and three sites that are overhunted but structurally intact, provides a unique opportunity to assess the differential effects of these defaunation drivers on faunal communities at landscape scales.

## Results

### Functional extinction

**Fig. 1:**
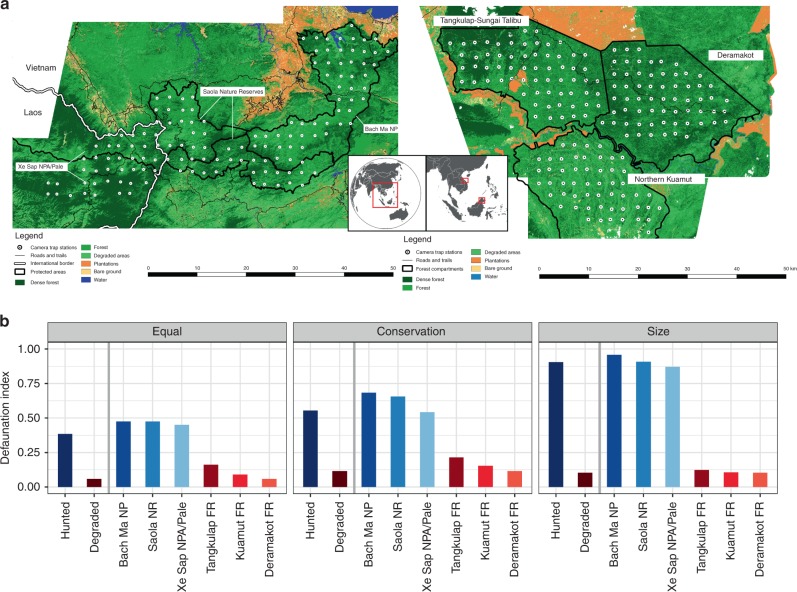
**a** Study sites in Vietnam/Laos (hunted) and Malaysian Borneo (degraded). **b** Historical defaunation indexes for hunted and degraded sites. Defaunation values were calculated using a measure of functional extinction, defined as species recorded in <2.5% of camera trap stations per site. Larger and more threatened species have higher levels of functional extinction. Species importance is weighted in three ways: all species given equal importance (equal), based on conservation status (conservation), and based on species average body size raised to the power of 3/4 (size)

For the historical defaunation analysis, we defined species as functionally extinct if they were recorded in <2.5% of the total camera-trap locations in a study site (see Methods for more details). Using the defaunation index (see Methods), we found that the three hunted sites have functionally lost a considerable proportion of their terrestrial mammal and bird community (*D*_*equal*_ Bach Ma NP = 0.48, *D*_*equal*_ Saola NRs = 0.48, *D*_*equal*_ Xe Sap/Palé = 0.45), whereas functional extinction rates were low in all degraded sites (*D*_*equal*_ Deramakot FR = 0.06, *D*_*equal*_ Tangkulap FR = 0.16, *D*_*equal*_ Kuamut FR = 0.09) (Fig. 1). Functional extinction levels were substantially higher for threatened and larger species in the hunted sites (*D*_*threatened*_ Bach Ma NP = 0.68, *D*_*threatened*_ Saola NRs = 0.66, *D*_*threatened*_ Xe Sap/Palé = 0.54; *D*_*size*_ Bach Ma NP = 0.96, *D*_*size*_ Saola NRs = 0.91, *D*_*size*_ Xe Sap/Palé = 0.87) but there was little difference when these species weightings were applied to the faunal community in the degraded sites (*D*_*threatened*_ Deramakot FR = 0.12, *D*_*threatened*_ Tangkulap FR = 0.21, *D*_*threatened*_ Kuamut FR = 0.15; *D*_*size*_ Deramakot = 0.10, *D*_*size*_ Tangkulap FR = 0.12, *D*_*size*_ Kuamut FR = 0.11). The same patterns were evident when the three hunted are combined as one site and three degraded sites are combined and evaluated as one site (*D*_*equal*_ hunted = 0.38, *D*_*threatened*_ hunted = 0.55, *D*_*size*_ hunted = 0.91; *D*_*equal*_ degraded = 0.06, *D*_*threatened*_ degraded = 0.12, *D*_*size*_ degraded = 0.10). Defaunation levels also showed distinct patterns among the individual sites. Amongst the hunted sites, under equal species weighting, Bach Ma NP and the Saola NRs had the highest defaunation values, followed by Xe Sap/Palé. When species were weighted to reflect conservation priority and size, Bach Ma NP has the highest defaunation, followed by the Saola NRs, followed by Xe Sap/Palé. Defaunation levels in the degraded sites showed a consistent pattern, independent of the species weighting. Amongst the degraded sites, Tangkulap FR had the highest defaunation values, followed by the most degraded site (Kuamut FR), followed by the least degraded site (Deramakot FR). In the degraded landscape only four species (11.1%) were considered functionally extinct. However, in the hunted sites 25 (55.6%) species were considered functionally extinct. We found no evidence in the hunted sites of large carnivores, megaherbivores, or a substantial component of the galliform community that would have historically existed in the area (Supplementary Table 1).

### Species occurrence patterns

**Fig. 2:**
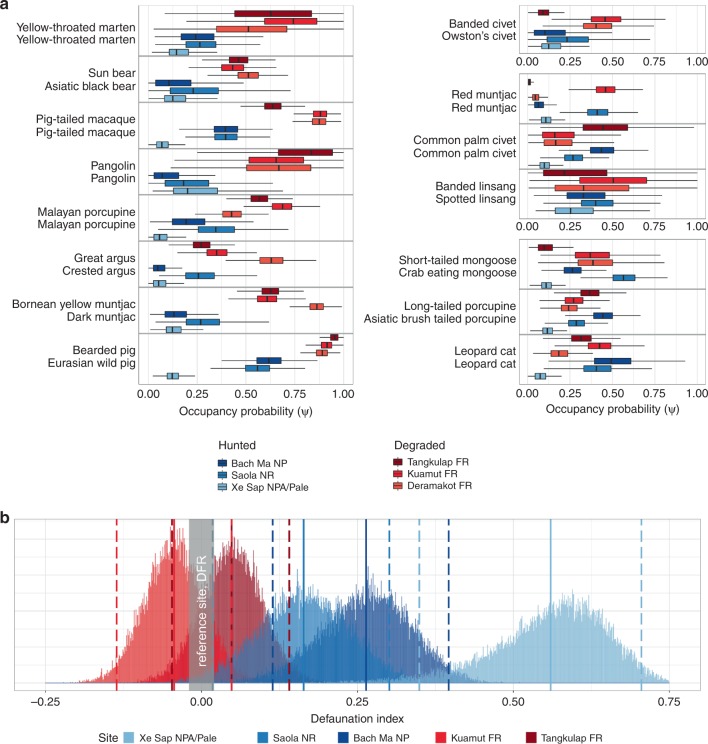
**a** Bayesian community occupancy estimates for 15 mammal and terrestrial bird species or species pairs for each of the six study sites. Species occupancy estimates (mean and 95% BCI) from the hunted and degraded sites. Species occupancy estimates for the hunted sites are shown in blue colors. Species occupancy estimates for the degraded sites are shown in red colors. Average occupancy was higher in the degraded sites than in the hunted sites for most species pairs (lefthand panel). **b** Occupancy-based defaunation index for 15 mammal and terrestrial bird species or species pairs in two hunted and three degraded sites. The degraded but non-hunted site (Deramakot FR) is used as a reference site (zero defaunation). The occupancy-based defaunation index is higher for the hunted sites than the degraded sites. Solid lines represent mean values; dotted lines represent 95% Bayesian credible intervals

To assess and compare the impacts of defaunation on species occurrence, we used terrestrial mammal and large galliform species or species pairs that still occurred in both landscapes. In total 15 species or species pairs were found to still occur in both landscapes and thus could be included in the analysis. Species pairs were chosen based on taxonomic and ecological similarities (Supplementary Table 2). We used Bayesian community occupancy models to estimate and compare probabilities of occurrence between the hunted and degraded landscapes. We included two covariates in the analyses: village density (*x*_*village*_) and canopy closure (*x*_*canopy*_). We found that estimated occupancies were lower in all three hunted compared to the three degraded sites for eight of the 15 species pairs (Fig. 2a; Supplementary Table 3), whereas there were no cases where the occupancies in all of the hunted sites were higher than the occupancies in all of the degraded sites. One species pair showed higher occupancies in two of the three degrades sites, with estimated occupancy for the third degraded site similar to the three hunted sites. Three species pairs had occupancies that were similar among the hunted and degraded sites. An additional three species pairs also showed occupancies that were similar among the hunted and degraded sites, but with one hunted site having a higher estimated occupancy than any of the degraded sites. Four of the six species with comparable occupancies among hunted and degraded sites were generalist mesocarnivores.

The effects of defaunation on species occurrence was also assessed using an occupancy-based defaunation index, calculated using the posterior occupancy estimates from the Bayesian community occupancy model for the 15 species pairs, and using the least degraded and non-hunted site (Deramakot FR) as the reference assemblage (*D*_*occupancy*_ *=* 0). Defaunation values were higher for the three hunted sites (*D*_*occupancy*_ Bach Ma NP = 0.26 ± 0.07, *D*_*occupancy*_ Saola NRs = 0.16 ± 0.07, *D*_*occupancy*_ Xe Sap/Palé = 0.56 ± 0.09) than the two degraded sites (*D*_*occupancy*_ Tangkulap FR = 0.05 ± 0.05, *D*_*occupancy*_ Kuamut FR = −0.04 ± 0.05) and the reference site (Fig. 2b). Among the hunted sites, the most defaunated site was Xe Sap/Palé, and the least defaunated site were the Saola NRs. Among the degraded sites Tangkulap FR had a higher defaunation value than the reference site (*D*_*occupancy*_ Tangkulap FR = 0.05 ± 0.05), as expected by the higher degradation. However, the most degraded site Kuamut FR had a lower defaunation value (*D*_*occupancy*_ Kuamut FR = −0.04 ± 0.05) than the reference site. The negative defaunation value for Kuamut FR indicates that estimated occupancies are higher in the more degraded site than in the reference site for these 15 species.

### Drivers of species occurrence

**Fig. 3:**
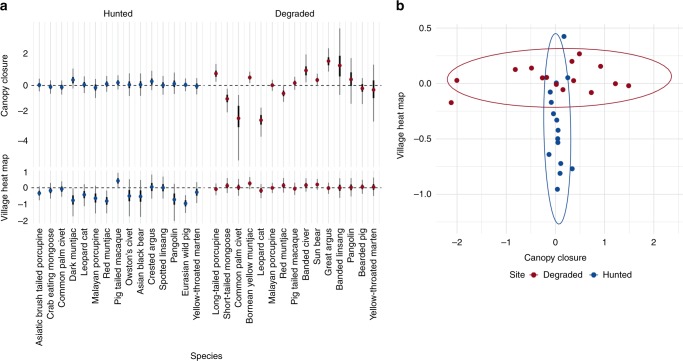
**a** Effect sizes for two covariates, canopy closure and village density, on Bayesian occupancy model results for 15 species or species pairs in the hunted and degraded sites. Canopy closure is used as a proxy for habitat degradation, and village density is used as a proxy for hunting pressure. 75% BCIs are shown with a thick black line, 95% BCIs are shown with a thin black line. **b** Mean covariate effect sizes for each species pair plotted against each other. The ellipses represent the 95% data ellipses

To assess the third hierarchical level of defaunation, we investigated how anthropogenic and habitat-based factors influence species occurrence. We evaluated covariate (*x*_*village*_ and *x*_*canopy*_) effect sizes for the 15 species pairs within the Bayesian community occupancy framework. For most species in the degraded landscape occurrence was strongly influenced by the habitat-based covariate (nine species with 95% BCI’s that do not overlap zero for *x*_*canopy*_) (Fig. 3a, Supplementary Table 4). In contrast, the environmental driver had minimal impact on species occurrence for species in the hunted landscape (no species with non-overlapping effect size 95% BCIs, two species with non-overlapping 75% BCIs for *x*_*canopy*_). Species occurrence in the hunted landscape was strongly associated with the anthropogenic covariate (three species with non-overlapping effect size 95% BCI’s, seven species with non-overlapping 75% BCIs for *x*_*village*_) but had minimal effect on species occupancies in the degraded sites (no species with non-overlapping effect size 95% BCIs, three species with non-overlapping 75% BCIs for *x*_*village*_). To assess if the greater response to *x*_*canopy*_ in the degraded sites was due to more variability in the canopy closure covariate in Malaysian Borneo compared to the Annamites sites, we subsetted the canopy closure sampling locations for the degraded sites so that the mean and variation was similar to the hunted sites, (Supplementary Fig. 1) and ran a community occupancy model for the 15 species. Our results show a similar response for canopy closure, even with the subsetted data (Supplementary Fig. 2).

When the effect sizes for *x*_*village*_ and *x*_*canopy*_ are plotted against each other, canopy closure showed a strong effect on species occurrence for the degraded sites, but weak effect in the hunted sites. Village density shows a strong effect on species occurrence for the hunted sites, with little impact on occupancies for the degraded sites (Fig. 3b). Canopy closure shows both positive and negative effects on species occurrence in the degraded sites. In contrast, village density shows a persistent negative impact on species occurrence in the hunted sites, with only one species, Northern pig-tailed macaque *Macaca leonina*, having higher estimated occupancy in areas closer to villages.

## Discussion

Our results provide insight into the differential impacts of moderate habitat degradation and intensive, indiscriminate hunting on tropical mammal and bird communities at multiple hierarchical levels of the defaunation process. At the most fundamental level habitat degradation and indiscriminate hunting drive species extinctions. We found that both defaunation drivers resulted in functional extinctions in our study sites, but that the relative impact of these drivers differed substantially. Higher defaunation in the hunted sites suggests that, within the context of species loss in tropical forests, widespread indiscriminate hunting is unsustainable and may be a more severe short-term threat than the moderate levels of habitat degradation considered in this study. At first these results contradict conventional thinking on the consequences of these two drivers, because hunting is often considered to impact a few target species, whereas degradation is seen to impact all species within a community. However, it is important to note that, in contrast to earlier studies that predominantly assessed the effects of gun-hunting on faunal communities^26–28^, our study investigated the consequences of indiscriminate snaring. Our findings support earlier observations that show that snaring causes declines in a wide range of ground-dwelling vertebrates^41^. To date, large-scale conservation initiatives in tropical countries have predominantly focused on habitat conservation. Our results suggest that, to protect tropical terrestrial mammal and bird communities, a paradigm shift may be warranted, in which these initiatives focus as much on addressing unsustainable hunting as on activities that result in moderate levels of habitat degradation.

We also found that functional extinction rates were higher in the hunted sites for both threatened and larger species compared to the equal species weighting, but that there was little difference in the degraded sites. Greater susceptibility of conservation-priority and larger species further underscores the potentially greater negative impact of hunting compared to degradation. Threatened species are important from a conservation perspective, and may serve as flagships for wider conservation initiatives^52^. Larger mammals often have greater impacts on ecosystems through predation and herbivory, and their extinction can cause fundamental changes in ecosystem functions^53^. We note that, because we gave all species a scaled body mass weighting, the body size bias in the size-weighted defaunation index is a function of the total body mass of the community, and should not be caused solely by the loss of the largest mammal species. However, to ensure that the loss of the largest mammalian species (elephant *Elephas maximus*, gaur *Bos gaurus*) did not disproportionally skew our results, we also tested a ranked order weighting, which gave results similar to the weighted analyses (Supplementary Fig. 3). The loss of either threatened species or larger mammals undermines conservation and sustainability-based objectives. The fact that our degraded sites experienced relatively low levels of functional extinction highlights the potential conservation value of secondary forests. This finding is consistent with previous studies that have shown that logged secondary forests can still retain substantial components of their original faunal assemblage^54,55^. However, we note that the conservation value of degraded areas would be low if these areas have experienced heavy hunting pressure, which is a likely scenario given the fact that hunting and logging are often closely linked^55^. It is, therefore, possible that the low levels of functional extinction that we documented in our degraded sites represent a best-case scenario. Nonetheless, we believe that our results provide an optimistic assessment for the potential of degraded forests to contribute to the maintenance of tropical biodiversity. In some situations, the financial revenues from sustainable logging might provide additional resources that could help protect forests from hunting.

Defaunation also decreases species abundance and distribution. We found that most species pairs that occurred in both hunted and degraded sites showed lower occupancy in the hunted sites, and that occupancy-based defaunation values for these species were several times greater in sites that were subject to hunting pressure (Fig. 2). Because decreases in species occupancy often lead to local extinctions, this finding has obvious conservation implications, especially for range-restricted species. Annamite striped rabbit occupancy is already so low in Bach Ma NP that, without immediate reduction in snaring pressure, the species will soon become locally extinct in the protected area^56^. The loss of a range-restricted Annamite endemic from one of the few areas where the species has been confirmed^57^ is a poignant reminder of the link between declining occurrence and extinction. Decreases in abundance and distribution can also have less obvious, more systemic consequences. Declines in species occurrence can degrade ecological interactions, leading to fundamental changes in ecosystem processes^58^. Previous studies, for example, have shown that ungulate declines may reduce seed dispersal, which in turn impacts vegetation communities and forest structure^59^. Such changes in forest structure across large areas of the tropics have numerous implications. Recently, Bello et al.^60^ showed that faunal declines may fundamentally erode carbon storage capacities in tropical rainforests. Thus, increasing conservation emphasis on overhunting may not only prevent functional extinctions, but also preserve the ecological integrity of tropical forests.

We found that four of the six species with comparable occupancies among hunted and degraded sites were mesocarnivores (Fig. 2). We were surprised to find that these mesocarnivore occupancies were similar between heavily defaunated and more intact sites. One explanation for this finding is that mesocarnivores embody traits that make them more resilient to hunting pressure. When compared to apex predators, mesocarnivores tend to have more flexible dietary requirements, often preying on small mammals or invertebrates that are not utilized by carnivores at higher trophic levels^61^. Furthermore, some mesocarnivores are also highly omnivorous. For example, the common palm civet *Paradoxurus hermaphroditus*, one of the most abundant mesocarnivores in our hunted sites, has been known to subsist on fruit^62^. These generalist traits may make mesocarnivore species less susceptible to declines due to hunting. An alternative explanation is that small carnivores have increased in abundance and distribution in our hunted sites through a mesopredator release mechanism^63,64^. Historically, our hunted sites would have included a range of top carnivore species – including tiger *Panthera tigris*, leopard *Panthera pardus*, dhole *Canis alpinus*, clouded leopard *Neofelis nebulosa*, and Asian golden cat *Catopuma temminckii* – all of which are now locally extirpated or present at functionally extinct levels (Supplementary Table 1). Studies in other terrestrial ecosystems have shown that the decline of apex predators reduces both direct and indirect competition on mesocarnivores, often resulting in unnaturally high densities for these species^65^. Although our findings give some insight into the persistence of mesopredators in faunally impoverished systems, we note that more in-depth studies are needed to assess the extent to which defaunation drivers benefit this species group, and therefore contribute to the biotic homogenization^66^ of tropical faunal communities.

Surprisingly, we found that occupancy-based defaunation values were lower in our most degraded site (Kuamut FR) than in the least degraded reference site (Deramakot FR), indicating that overall occupancy for these 15 species increased with degradation. We believe this result can be explained by the fact that our analysis was limited to species that were recorded in both the hunted and degraded landscapes. Many of the species present in the hunted sites were highly adaptable generalist mammal species known to be resilient to anthropogenic pressures. Our comparative analysis may, therefore, be biased towards more generalist species that tend to be more resilient to both defaunation drivers. To test this assumption we ran occupancy models for the entire suite of mammal and bird species in the degraded sites (in total 32 species instead of the subset of 15) and the results clearly show that, while species-specific responses vary, habitat degradation negatively impacts the faunal community as a whole, and our reference site (Deramakot FR) had on average the highest species occupancies and thus the lowest defaunation values (Supplementary Fig. 4).

Defaunation drivers also impact the underlying factors that influence species distribution. We found that anthropogenic and habitat-based covariates differed in their importance in explaining species occurrence patterns in our study sites (Fig. 3). Understanding the factors that influence species occurrence is important for numerous tools used in conservation science. For example, in recent years species distribution modeling has become an integral component of conservation planning^67,68^. To date, the field of species distribution modeling has largely focused on the use of ecological variables to predict distribution^69^, with less emphasis on the inclusion of anthropogenic covariates that reflect spatial variation in hunting pressure (but see Lippitt et al.^70^). In areas characterized by hunting-driven declines, spatial prioritizations built upon species distribution models that only use ecological variables may poorly represent actual biodiversity patterns, which can in turn lead conservation stakeholders to misallocate limited conservation resources. We acknowledge that finding proxies that accurately capture hunting pressure may be challenging, as hunting pressure itself is a complex phenomenon resulting from various socio-economic and cultural influences. However, we are optimistic that recent advances in statistical modeling and earth observation science^71^ will provide new opportunities for the development of increasingly sophisticated anthropogenic covariates for use in species distribution models. In the Annamites, novel approaches are already being developed that take into account hunter accessibility across both spatial and temporal dimensions^72^. We hope that our findings encourage further developments into this field as hunting is a key driver of species occurrences and therefore should not be neglected.

Our comparative analyses provide new insights into the effects of moderate habitat degradation and indiscriminate hunting on tropical mammal and bird communities. However, we also recognize that, because these defaunation drivers are the result of complex and often locally-specific processes, further research is needed to provide a more holistic understanding of their impacts. We first acknowledge that data from additional sites is needed to obtain a more holistic picture of how different defaunation drivers impact faunal communities, especially as our three study sites in the two landscapes were adjacent to one another. Although we believe that our landscape approach, with study sites over 300–400 km^2^ in size (much larger than the home range of any species included in this analysis), make our study less vulnerable to the spatial effects that could arise from surveying adjacent sites, our sites might not be spatially independent in the strictest sense of the term. Second, we recommend that future landscape-scale systematic camera-trapping include areas subject to more extreme levels of habitat degradation. Although our most degraded study sites had undergone intensive conventional logging, none of our study areas had been clear-cut. Disturbance levels in these sites are therefore at the moderate, rather than severe, end of the degradation spectrum. Although some studies have assessed faunal communities in degraded areas, most have been conducted over relatively small spatial scales^20^, failed to account for imperfect detection probabilities^73^, or used meta-analyses that rely on datasets that cover large spatial extents but may not be well-suited for in-depth analyses^21^. Additional standardized surveys using occupancy-based approaches may reveal a bleaker picture of degradation-driven declines than we found. We caution that, until such studies are conducted, our results should only be interpreted within the context of moderate levels of habitat degradation. A similar point can be made with regard to hunting pressure. Because indiscriminate snaring impacts a wide range of taxa^41^, it is likely that areas subject to more selective gun-hunting will not show the same degree, or species-specific patterns, of faunal decline. Our findings are therefore most applicable to other areas where nonselective methods of wildlife exploitation predominate. We also recognize that the magnitude of snaring in our sites is exceptionally high, and that future studies in areas under less extreme snaring pressure may provide a more nuanced perspective into hunting-driven defaunation. However, here we point out that, because industrial-scale snaring is rapidly expanding across the tropics, especially in Southeast Asia^41^, we believe that our findings may be directly relevant to an increasing number of tropical regions in the near future.

Given future population projections^74^ and road expansion in developing countries^75^, tropical rainforests will be subjected to ever-increasing levels of degradation and exploitation. Pantropical defaunation can only be prevented if conservation stakeholders develop effective conservation solutions to address these threats in the most efficient way. But determining how best to implement these solutions with limited conservation resources remains a challenge. Our results show that, while both defaunation drivers negatively impact tropical faunal communities, unsustainable hunting practices such as the widespread, indiscriminate hunting examined here may be the more severe short-term threat for terrestrial mammal and bird species. We suggest that conservation strategies that seek to protect tropical faunal communities may benefit by focusing on actions that mitigate against unsustainable hunting, rather than moderate levels of habitat degradation. Because unsustainable hunting is linked to such a complex range of social, economic, and cultural issues, developing strategies to address this challenge may require new ways of thinking. Ultimately, maintaining healthy tropical faunal communities is in the best interest of conservationists that want to protect biodiversity, national governments that seek to maintain ecosystem services, and local communities that rely on having access to sustainable forest resources. Bringing these diverse stakeholders together may help in the development of novel conservation approaches.

## Methods

### Study areas and design

We used systematic camera trapping to collect data on the ground-dwelling mammal and bird communities in six study areas in Southeast Asia. Stations were spaced approximately 2.5 kilometers apart (Annamites: x̅ = 2.47 ± 0.233 km; Malaysian Borneo: x̅ = 2.46 ± 0.220 km, Fig. 1a). At each station two white-flash camera traps (Reconyx® Hyperfire Professional PC850; Reconyx, Holmen, USA) were set facing in different directions. Cameras were placed along trails, ridgelines, and water sources to maximize detections of mammals and ground-dwelling birds. All cameras were placed 20–40 cm above the ground, were operational 24 h per day, and were left in the field for a minimum of 60 days.

Systematic camera trapping in the Annamites was conducted between November 2014 and December 2016 (Supplementary Table 5) in a continuous forest across Vietnam and Laos. In total the survey areas cover more than 1000 km^2^ of broadleaf evergreen lowland and upland dipterocarp tropical rainforest, split into three study sites. In Vietnam, we surveyed: Bach Ma NP (~340 km^2^) and the Hue and Quang Nam SNRs (together approximately 275 km^2^). In Laos, we surveyed the eastern section of Xe Sap and the adjacent Palé area (together ~300 km^2^). The Palé area is categorized as a watershed protection forest. The two study sites in Vietnam are surrounded by densely-populated human-modified areas that contain permanent settlements, plantations, and agricultural fields. By contrast, the Lao site does not contain extensive human-modified areas, and population density is low. However, the eastern part of Xe Sap NPA and the Palé areas are heavily utilized by Vietnamese poachers and gold mining operations^49^. Poaching, primarily accomplished by the setting of wire snares, occurs in all sites. Because snaring pressure is related to a complex set of factors, further complicated by different management regimes among the sites, we did not make *a priori* assumptions into the underlying gradient of hunting pressure across the sites.

Systematic camera trapping in Malaysian Borneo was conducted between October 2014 and July 2016 (Supplementary Table 5). We used the same survey design as in the Annamites, with two camera traps set in different directions at each station, and stations spaced approximately 2.5 km apart. As with the study sites in the Annamites, the Malaysian Borneo sites contain wet evergreen lowland and upland dipterocarp tropical rainforest. We surveyed three logging concessions that form a contiguous forest block: Deramakot FR, Tangkulap-Sungai Talibu FR, and Northern Kuamut FR. The concessions have been subjected to varying levels of habitat degradation from both past and current logging. From the 1950s to 1989, Deramakot FR (~550 km^2^) was licensed to a private logging company^44–46^. In 1989, management of the forest passed to the Sabah Forestry Department and logging activities stopped. Reduced-impact logging was initiated in 1995^44–46^. Deramakot FR uses a 40-year logging cycle to allow forests to regenerate before harvest^44–46^. In 1997, the concession obtained Forest Stewardship Council (FSC) certification, Dermakot FR is promoted as the flagship of the Sabah Forestry Department for sustainable forest management^76^. From the 1970s to 2002, Tangkulap FR (approximately 501 km^2^) was managed by a private logging company, and was repeatedly logged using conventional logging techniques^46^. Logging stopped in 2001, and in 2011 the reserve received FSC certification^46^. Although the forest has regenerated during this interim, it remains moderately degraded due to the intensity of past conventional logging activities^46^. In 2015, Tangkulap FR was reclassified as a protected area, except for 2000 ha of industrial timber plantation area that remains as production forest. Kuamut FR (~695 km^2^) was intensively logged using conventional techniques between 2004 and 2012, and the forest is highly degraded^44–46^. In 2016, Kuamut FR was reclassified as a class 1 protected area in Sabah. Overall, Kuamut FR is the most degraded of the three areas, followed by Tangulap FR, then Deramakot FR (44–46, Supplementary Fig. 5). There are a small number of villages around the periphery of the three sites. However, there is little evidence that villagers engage in routine hunting inside the study sites^46^.

To avoid any confounding effects due to greater visibility around camera-trap stations, our data collection procedure was standardized prior to field work. We have developed detailed field protocols for camera-trap setup (see Abrams et al.^77^). The camera-trap setup protocols include the removal of vegetation to ensure that the area surveyed by the camera-trap is comparable for all sampling stations and, therefore, among the different study sites. We note that, in contrast to previous studies that combine data *post hoc*, our study was conceptually planned as a part of one project, with regular interactions between the field teams before and during the fieldwork to ensure the standardization of data collection procedures. Finally, because detectability can also be influenced local factors, such as different movements or abundances, species detection rates can vary between hunted (Annamites) and degraded (Malaysian Borneo) study sites. We, therefore, modeled detectability differently between the hunted and degraded sites (see full model description below).

### Occupancy covariates

We modeled species occurrence using occupancy models that account for imperfect detection^51^. We used covariates to assess the factors that influence species occurrence, and to improve model fit. We included two covariates in our models: canopy closure and village density. We expect that canopy closure would have more influence on species occurrence in the three degraded sites, and that village density would have more influence in the three hunted sites.

We used canopy closure as a measure of forest degradation. We consider higher canopy closure values to indicate more intact forest, and lower values to indicate more degraded forest^78^. Canopy closure was assessed in situ. In the field, we established a 20 × 20 m grid at each station, with the centerpoint halfway between the two camera traps. The grid was positioned along the north-south, east-west axes. We used a handheld GPS (Garmin® model 62sc, Garmin Ltd, 107 Canton of Schaffhausen, Switzerland) to take canopy photographs at the centerpoint and the corners of the grid (northwest, northeast, southeast, and southwest). Coordinates were recorded for each photograph, and the Waypoint Averaging function was used to minimize GPS error. Canopy photographs were manually converted to black and white using the open source GNU Image Manipulation Program^79^, producing a raster file with black areas representing vegetation and white areas representing open sky. We then used R *v. 3.4.0*^80^ to calculate percentage canopy closure for each image. Finally, canopy closure values were averaged across the five rasters to give a single mean canopy closure value for each camera-trap station. Detailed information on canopy data collection protocols can be found in Abrams et al.^77^ Canopy closure values are given as a percentage, with higher values indicating a more intact canopy, and more structurally intact forest. The canopy closure values show that the sites in Malaysian Borneo are more degraded than the sites in the Annamites (x̅_Borneo_ = 0.77 ± 0.2, x̅_Annamites_ = 0.83 ± 0.05). Among the degraded sites in Malaysian Borneo, canopy closure values were highest for Deramakot FR (x̅_Deramakot_ = 0.84 ± 0.12), followed by Tangkulap FR (x̅_Tangkulap_ = 0.73 ± 0.22), followed by Kuamut (x̅_Kuamut_ = 0.71 ± 0.23). The canopy closure values for the Malaysian Borneo sites reflect the degradation levels that would be expected based both on historical logging patterns and observations made in situ. The three sites in the Annamites had similar canopy closure values (Supplementary Fig. 4).

We used village density as a proxy for hunting at the local scale. Hunting pressure is often related to accessibility^4^, and several studies in other tropical regions have demonstrated a defaunation gradient around local villages^81–83^. To calculate village density we first created a heatmap in QGIS *v. 2.18.9*^84^ using a village shapefile as the input point layer. We used the default quartic kernel decay function and set the radius to 15 km. The village density radius was chosen so that all individual sampling stations in our study landscape fell within the hunting halo in the final heatmap. Observations while conducting fieldwork in the Annamites indicate that all camera-trap stations, even those in the most remote areas of the Palé area, were subject to some level of hunting pressure, as evidenced by the presence of wire snares. To be consistent among sites, we also used the same village density radius for the study sites in Malaysian Borneo. After creating the heatmap, we used the extract function in the *raster* package^85^ to obtain village density values for each station. The village density covariate is unitless, with higher values indicating areas closer to a higher number of villages, and lower values indicating areas that are more remote. Consistent with observations made in situ, in the Annamites, Bach Ma NP had the highest density of surrounding villages, followed by the Saola NRs, followed by Xe Sap/Palé (Supplementary Fig. 4). The sites in Malaysian Borneo had low village density values, reflecting the low number of villages in their vicinity.

### Historical defaunation index

We used the defaunation index proposed by Giacomini and Galetti^51^ to calculate historical defaunation for each study area. This defaunation index is a weighted measure of dissimilarity between an assemblage of interest and a reference assemblage representing a historical or less disturbed site. The defaunation index is given by the equation:

$$D\left( {r,f} \right) = \frac{{\mathop {\sum }\nolimits_{k = 1}^S \omega _k\left( {N_{k,r} - N_{k,f}} \right)}}{{\mathop {\sum }\nolimits_{k = 1}^S \omega _k\left( {N_{k,r} + N_{k,f}} \right)}}$$

where *D* is the index of defaunation of focal assemblage *f* with respect to a reference assemblage *r*; *S* is the total number of species in the focal (*f*) and reference (*r*) assemblages; *k* is the identification of a species; *N*_*k,r*_ is presence or absence of species *k* in the reference assemblage; *N*_*k,f*_ is presence or absence of species *k* in the focal assemblage; and *ω*_*k*_ is the weight assigned to species *k*. When comparing a more defaunated assemblage to a reference assemblage, *D* ranges from 0 to 1. It is also important to note that *D* can assume negative values if the focal assemblage is less defaunated than the reference assemblage. It is, therefore, possible for *D* to range from −1 to 1, with positive values indicating more defaunation, and negative values indicating less defaunation.

To construct the historical reference assemblage, we used IUCN range maps to document ground-dwelling mammal and terrestrial bird species that historically occurred in each study area. We included mammal and terrestrial bird species >500 g in our analyses for two reasons. First, smaller species are unlikely to be impacted by snaring^56^. Second, many smaller mammals (rodents, squirrels) and birds (partridges) are difficult to identify to species level using camera trap photographs. We excluded highly arboreal species in our analysis—for example, the red-shanked douc langur *Pygathrix nemaeus* in the Annamites, the dusky langur *Presbytis rubicunda* in Malaysian Borneo, and all large Sciuridae from both landscapes—as these species are unlikely to be reliably detected by camera-traps placed at ground level. We also excluded riverine habitat specialist species, for example all otter species (*Lutra* spp., *Lutrogale perspicillata*, and *Aonyx cinerea*), because the majority of our camera stations were not located on streams or rivers and, as a result, we believe that it is possible that our study would not have recorded these species even if present. Finally, we did not include weasel species for either study area (*Mustela kathiah* in the Annamites, *Mustela nudipes* in Malaysian Borneo), as these species may be routinely under-recorded by camera-trapping^86–88^. Species that could not be confidently identified to species level using camera-trap images were grouped at the genus level. In the Annamites, Chinese pangolin *Manis pentadactyla* and Sunda pangolin *M. javanica* were grouped as *Manis* spp., and the large-toothed ferret badger *Melogale personata* and small-toothed ferret badger *M. moschata* were grouped as *Melogale* spp. We also grouped all images of Annamite dark muntjac *Muntiacus rooseveltorum/truongsonensis*, as the taxonomy for this species complex is currently unresolved^89,90^. In Malaysian Borneo, the greater mousedeer *Tragulus napu* and lesser mousedeer *Tragulus kanchil* were grouped as *Tragulus* spp. The final historical reference assemblages provide a validated list of terrestrial mammal and bird species that historically occurred at each site (Supplementary Table 1).

For the historical defaunation analysis, we compiled a list of species that were recorded in <2.5% of our total camera-trap locations at each study site, and considered these species as functionally extinct. We chose to use a measure of functional extinction as defined by an occupancy-based metric, rather than a measure of complete extinction defined by species recorded or not recorded during our study, for two reasons. First, even if a species was not recorded during our surveys, it would be incorrect to infer species absence. Second, using a functionally extinct definition allows for the possibility that a species may be present but not in numbers that constitute an ecologically functional population. Because the number of stations was different between the sites in the Annamites and Malaysian Borneo, we decided to use 2.5% of all stations instead of a fixed number of minimum stations. 2.5% of total stations represent two stations for Bach Ma NP, two stations for the Saola NRs, and one station for Xe Sap/Palé. 2.5% of stations represent two stations for Deramakot FR, two stations for Tangkulap FR, and two stations for Kuamut FR. We believe that this low number of stations is a conservative estimate for a species to exist in the landscape and remain ecologically functional. Therefore, the final current species assemblage, therefore, gives a conservative estimate of functionally extinct mammals and terrestrial birds in our study sites (Supplementary Table 1).

To assign species weights in the historical defaunation index, we followed the methods presented by Giacomini and Galetti^50^. We used three species weights: equal weighting, threatened status as an indication of conservation priority, and average body mass^50^. We derived threat status by assigning values for each species using *The IUCN Red List of Threatened Species* (assessed as of February 1st, 2019). Weights were given as follows: Least Concern = 1; Near Threatened = 2; Vulnerable = 3; Endangered = 4; and Critically Endangered = 5. We did not have any species in our dataset classified as Extinct or Extinct in the Wild. Two species from the sites in the Annamites (Annamite dark muntjac species complex *Muntiacus rooseveltorum/truongsonensis* and Annamite striped rabbit *Nesolagus timminsi*) were listed as Data Deficient. We assigned these two species a mean value of 2.5. We also assigned species weights based on average body mass. Average body mass was taken from natural history books and regional field guides^32,33,91^. If this information was not available for a species, we used the average body mass for a similar species as an approximation. Following Giacomini and Galetti^50^, we raised the body mass to the power of ¾ to better reflect species functions based on body size (Supplementary Table 1).

### Community occupancy analysis

We adopted the hierarchical formulation of occupancy models by Royle and Dorazio^51^ and extended this to a community occupancy model by linking the species-specific models by assuming that species-specific parameters come from a common underlying distribution, governed by community hyperparameters. To assess the impacts of defaunation on species occurrence, we ran community occupancy models for 15 phylogenetically closely-related terrestrial mammal and galliform species or species pairs. Species pairs were restricted to species that occur in both the Annamites and Malaysian Borneo. For example, we could not use serow *Capricornis milneedwardsii* in the analyses because the species occurs in the Annamites but not in Borneo, and we could not include the Bornean orangutan *Pongo pygmaeus* because the species occurs in Borneo but not in the Annamites. Similarly, we could not include binturong *Arctictis binturong* because, while the species range includes both Borneo and the Annamites, we did not record it in our surveys in the Annamites. All species pairs represent taxa that are approximately the same body size and in the same feeding guild (Supplementary Table 5). While we acknowledge that there may be site-specific differences in the ways in which species or species pairs respond to anthropogenic pressures, given the functional similarities between the pairs, we believe that responses are likely to be generally similar.

Our minimum camera-trapping period was 60 days (x̅ = 68.8 days) and spacing was approximately 2.5 km (see Study areas and design section above). We consider our trapping period to satisfy occupancy closure and independence assumptions^92^. To establish species encounter histories, we pooled camera-trap data into 10-day occasions, resulting in at least six sampling occasions for all stations, and determined for each site and occasion whether a given species was detected or not. A 10-day occasion length was chosen to maximize the number of occasions while simultaneously avoiding zero-inflation in the encounter history dataset.

We ran separate Bayesian community occupancy analyses for each study site. We modeled occupancy probability as having a species and site-specific random intercept. We modeled the effect on occupancy of two covariates: canopy closure (*x*_*canopy*_) and village density (*x*_*village*_). Covariate values were normalized. We accounted for varying camera-trapping effort within the 10-day occasion as the only covariate on detection probability (*p*). The full community occupancy model had the following parameterization:

$$z_{ij}\sim Bernoulli( {\psi _{ij}})$$

$$logit(\psi _{ij}) = \alpha _{i,site[j]} + \beta 1_i \ast \,{\mathrm{canopy}}_j + \beta 2_i \ast \,{\mathrm{village}}\,{\mathrm{density}}_j$$

$$\alpha _{i,site}\sim Normal(\mu _{\alpha,site} ,\sigma _{\alpha,site} )$$

$$\beta 1_i\sim Normal(\mu _{\beta 1},\,\sigma _{\beta 1})$$

$$\beta 2_i\sim Normal(\mu _{\beta 2},\,\sigma _{\beta 2})$$

$$y_{ijk}\sim Bernoulli\left( {p_{ijk}} \right)$$

$$logit(p_{ijk}) = \alpha.p_i + {\mathrm{\beta }}.e_i \ast \,{\mathrm{Effort}}_{jk}$$

$$\alpha.p_i\sim Normal(\mu.p_{\alpha.p},\,\sigma.p_{\alpha.p})$$

$$\beta.e_i\sim Normal(\mu.e_{\beta.e},\,\sigma.e_{\beta.e})$$

in which *z*_*ij*_ is the true occupancy state (0 or 1) of species *i* at camera trap station *j*; *ψ*_*ij*_ is the respective occupancy probability; *α* is the intercept of the logit-linear predictor of occupancy probability, and *β1* and *β2* are the coefficients for canopy coverage and village density, respectively. In our model, *y*_*ijk*_ are the observations (0 or 1) of species *i* at site *j* at occasion *k*; *p*_*ijk*_ are the respective detection probabilities; *α.p* is the intercept of the logit-linear predictor of detection probability, indexed by species; *β.e* is the effect camera-trap effort (effort) on detection probability given by the number of days a camera-trap was working within a 10-day occasion. Species-specific detection intercepts and *β.e* come from a normal distribution with community means (*µ.p*_*α.p*_, *µ.e*_*β.e*_) and standard deviations (*σ.p*_*α.p*_, *σ.e*_*β.e*_).

We implemented the model in a Bayesian framework using JAGS accessed through the R package *rjags v.4.7*^93^. We ran three parallel Markov chains with 250,000 iterations, of which we discarded 20,000 as burn-in, and we thinned the remaining iterations by 20 to make the output more manageable. We assessed chain convergence using the Gelman-Rubin statistic where values <1 indicated convergence^94^. We report results as posterior mean and standard deviation, and 95 and 75% Bayesian confidence intervals (95% BCI, 75% BCI, the 2.5% and 97.5%, and 12.5% and 87.5% percentiles of the posterior distribution, respectively; Supplementary Tables 3, 4).

### Occupancy-based defaunation index

We calculated an occupancy-based defaunation index for the 15 species and species pairs by incorporating the posterior distributions of occupancy probability into the defaunation index proposed by Giacomini and Galetti^50^. We used the distribution of occupancy estimates from Deramakot FR as our reference assemblage. We selected Deramakot FR as the reference because it is the least degraded site and is not subject to hunting pressure. We attach confidence intervals to each estimate of defaunation (*D*) by incorporating the uncertainty associated with the occupancy estimates (*ψ*). We used Monte Carlo sampling to construct the probability distribution of *D*. To do this we sampled random values from the posterior distributions of species-specific occupancy probabilities for all five sites for each species pair and used these values to calculate *D* where *N*_*k,r*_ and *N*_*k,f*_ are the occupancy of species *k* in the reference and focal assemblage, respectively. We repeated this procedure 30,000 times to generate a distribution of *D* values. 95% confidence intervals were calculated using the 2.5% and 97.5% percentiles of the distribution as confidence limits.

### Assessing covariate effect sizes

To assess the effects of covariates on estimated *ψ*, we derived covariate effect sizes using regression coefficients for each species from the community occupancy models. We calculated a community average value for *β* values of predictor variables. We scaled all covariates before analysis, with covariates scaled independently between the hunted and degraded sites, respectively. Numbers further from 0 indicate a stronger effect of the associated covariate. A positive effect size indicates that occupancy probability increases as the associated covariate increases.

### Reporting summary

Further information on research design is available in the Nature Research Reporting Summary linked to this article.

## Supplementary information

Supplementary information

Reporting Summary

## Data Availability

The data used in this study are not publicly archived because it contain information on the locations of Red Listed as well as hunted and traded species. However, all data in support of the findings of this study are available from the corresponding author by reasonable request.

## Code availability

All code in support of the findings of this study is available online at https://github.com/jabrams23/speciescomparison.

## References

[1] Butchart SH (2010). Global biodiversity: indicators of recent declines. Science.

[2] Bradshaw CJ, Sodhi NS, Brook BW (2009). Tropical turmoil: a biodiversity tragedy in progress. Front. Ecol. Environ..

[3] Achard F (2002). Determination of deforestation rates of the world’s humid tropical forests. Science.

[4] Benítez-López A (2017). The impact of hunting on tropical mammal and bird populations. Science.

[5] Dirzo R (2014). Defaunation in the Anthropocene. Science.

[6] Redford KH (1992). The empty forest. BioScience.

[7] Tilker A (2017). Saving the saola from extinction. Science.

[8] Galetti M, Dirzo R (2013). Ecological and evolutionary consequences of living in a defaunated world. Biol. Conserv..

[9] Galetti M (2013). Functional extinction of birds drives rapid evolutionary changes in seed size. Science.

[10] Nasi R, Taber A, Van Vliet N (2011). Empty forests, empty stomachs? Bushmeat and livelihoods in the Congo and Amazon Basins. Int. Forestry Rev..

[11] Young HS, McCauley DJ, Galetti M, Dirzo R (2016). Patterns, causes, and consequences of anthropocene defaunation. Annu. Rev. Ecol., Evolution, Syst..

[12] Margono BA, Potapov PV, Turubanova S, Stolle F, Hansen MC (2014). Primary forest cover loss in Indonesia over 2000–2012. Nat. Clim. Change.

[13] Imai N (2012). Effects of selective logging on tree species diversity and composition of Bornean tropical rain forests at different spatial scales. Plant Ecol..

[14] Hansen MC (2013). High-resolution global maps of 21st-century forest cover change. Science.

[15] Yue S, Brodie JF, Zipkin EF, Bernard H (2015). Oil palm plantations fail to support mammal diversity. Ecol. Appl..

[16] Alroy J (2017). Effects of habitat disturbance on tropical forest biodiversity. Proc. Natl Acad. Sci. USA.

[17] Brooks TM (2002). Habitat loss and extinction in the hotspots of biodiversity. Conserv. Biol..

[18] Cleary DF (2007). Bird species and traits associated with logged and unlogged forest in Borneo. Ecol. Appl..

[19] Meijaard E, Sheil D (2008). The persistence and conservation of Borneo’s mammals in lowland rain forests managed for timber: observations, overviews and opportunities. Ecol. Res..

[20] Brodie JF, Giordano AJ, Ambu L (2015). Differential responses of large mammals to logging and edge effects. Mamm. Biol..

[21] Burivalova Z, Şekercioğlu ÇH, Koh LP (2014). Thresholds of logging intensity to maintain tropical forest biodiversity. Curr. Biol..

[22] Bennett EL, Robinson JG (2000). Hunting of wildlife in tropical forests - implications for biodiversity and forest peoples. Environment Department working papers; no. 76. Biodiversity Series..

[23] Ripple WJ (2016). Bushmeat hunting and extinction risk to the world’s mammals. R. Soc. Open Sci..

[24] Cardillo M (2005). Multiple causes of high extinction risk in large mammal species. Science.

[25] Bogoni JA (2016). Landscape features lead to shifts in communities of medium-to large-bodied mammals in subtropical Atlantic Forest. J. Mammal..

[26] Cullen L, Bodmer ER, Valladares-Pádua C (2001). Ecological consequences of hunting in Atlantic forest patches, São Paulo, Brazil. Oryx.

[27] Di Bitetti MS, Paviolo A, Ferrari CA, De Angelo C, Di Blanco Y (2008). Differential responses to hunting in two sympatric species of brocket deer (Mazama americana and M. nana). Biotropica.

[28] Flesher KM, Laufer J (2013). Protecting wildlife in a heavily hunted biodiversity hotspot: a case study from the Atlantic Forest of Bahia, Brazil. Tropical Conserv. Sci..

[29] Brashares JS, Golden CD, Weinbaum KZ, Barrett CB, Okello GV (2011). Economic and geographic drivers of wildlife consumption in rural Africa. Proc. Natl. Acad. Sci. USA.

[30] Sodhi NS, Koh LP, Brook BW, Ng PK (2004). Southeast Asian biodiversity: an impending disaster. Trends Ecol. Evolution.

[31] De Bruyn, M., et al. Borneo and Indochina are major evolutionary hotspots for Southeast Asian biodiversity. *Syst. Biol.* 63, 879–901 (2014). 10.1093/sysbio/syu047 25070971

[32] Phillipps Q (2011). Phillipps’ field guide to the birds of Borneo..

[33] Phillipps, Q. Phillipps’ field guide to the mammals of Borneo and their ecology: Sabah, Sarawak, Brunei, and Kalimantan Vol. 105. (Princeton University Press, 2016)

[34] Baltzer, M. C., Dao, N. T. & Shore, R. G. Towards a vision for biodiversity conservation in the forests of the lower Mekong ecoregion complex: summary of the biological assessment for the Ecoregion Biodiversity Conservation Program in the forests of the lower Mekong ecoregion complex (WWF International, WWF Indochina, 2001).

[35] Van Dung V (1993). A new species of living bovid from Vietnam. Nature.

[36] Tuoc, D., Dung, V., Dawson, S., Arctander, P. & MacKinnon, J. Introduction of a new large mammal species in Viet Nam. Forest Inventory and Planning Institute (Science and Technology News, 4–13 March, Hanoi, Vietnam. 1994).

[37] Surridge AK, Timmins RJ, Hewitt GM, Bell DJ (1999). Striped rabbits in southeast Asia. Nature.

[38] Averianov AO, Abramov AV, Tikhonov AN (2000). A New Species of Nesolagus (Lagomorpha, Leporidae) from Vietnam with Osteological Description..

[39] Gaveau, D. L. et al. Four decades of forest persistence, clearance and logging on Borneo. *PLoS One*, 9, e101654 (2014). 10.1371/journal.pone.0101654 PMC4100734 25029192

[40] Bennet, E. L., Nyaoi, A. J., & Sompud, A. J. in *Hunting for sustainability in tropical forests*. (eds Robinson, J. & Bennett, E. L (Columbia University Press, 2000)

[41] Gray TN (2018). The wildlife snaring crisis: an insidious and pervasive threat to biodiversity in Southeast Asia. Biodivers. Conserv..

[42] Hearn AJ (2016). The first estimates of marbled cat *Pardofelis marmorata* population density from Bornean primary and selectively logged forest. PLoS One.

[43] Briceño-Méndez M, Naranjo EJ, Mandujano S, Altricher M, Reyna-Hurtado R (2016). Responses of two sympatric species of peccaries (*Tayassu pecari* and *Pecari tajacu*) to hunting in Calakmul, Mexico. Tropical Conserv. Sci..

[44] Ong, R., Langner, A., Imai, N., & Kitayama, K. in *Co-Benefits of Sustainable Forestry: Ecological Studies of a Certified Bornean Rain Forest*. (ed Kitayama, K.) (Springer Science & Business Media, 2012)

[45] Sollmann R (2017). (2017). Quantifying mammal biodiversity co‐benefits in certified tropical forests. Diversity Distrib..

[46] Brozovic R (2018). Effects of forest degradation on the moonrat *Echinosorex gymnura* in Sabah, Malaysian Borneo. Mamm. Biol..

[47] Meyfroidt P, Lambin EF (2008). Forest transition in Vietnam and its environmental impacts. Glob. Change Biol..

[48] Wilkinson N (2016). Report on effects of five years of snare removal patrols on snaring in the Thua Thien Hue - Quang Nam Saola Landscape: an analysis of data collected by Forest Guard patrols..

[49] Tilker A (2014). A survey of eastern areas of Xe Sap National Protected Area, Lao PDR, for Saola and other large ungulates; final report to Global Wildlife Conservation and the Saola Working Group..

[50] Giacomini HC, Galetti M (2013). An index for defaunation. Biol. Conserv..

[51] Dorazio RM, Royle JA (2005). Estimating size and composition of biological communities by modeling the occurrence of species. J. Am. Stat. Assoc..

[52] Caro TM, O’Doherty G (1999). On the use of surrogate species in conservation biology. Conserv. Biol..

[53] Sinclair ARE (2003). Mammal population regulation, keystone processes and ecosystem dynamics. Philos. Trans. R. Soc. Lond. Ser. B: Biol. Sci..

[54] Clark CJ, Poulsen JR, Malonga R, ELKAN PW (2009). Logging concessions can extend the conservation estate for Central African tropical forests. Conserv. Biol..

[55] Kleinschroth F, Healey JR (2017). Impacts of logging roads on tropical forests. Biotropica.

[56] Tilker, A. et al. A little-known endemic caught in the South-east Asian extinction crisis: The Annamite striped rabbit *Nesolagus timminsi*. *Oryx*, 1–10, 10.1017/S003060531800053 (2018).

[57] Tilker, A. et al. *Nesolagus timminsi*. The IUCN Red List of Threatened Species 2019: e.T41209A45181925 (2019)

[58] Valiente‐Banuet A (2015). Beyond species loss: the extinction of ecological interactions in a changing world. Funct. Ecol..

[59] Kurten EL (2013). Cascading effects of contemporaneous defaunation on tropical forest communities. Biol. Conserv..

[60] Bello C (2015). Defaunation affects carbon storage in tropical forests. Sci. Adv..

[61] Prugh LR (2009). The rise of the mesopredator. Bioscience.

[62] Nakashima Y, Inoue E, Inoue-Murayama M, Sukor JA (2010). High potential of a disturbance-tolerant frugivore, the common palm civet *Paradoxurus hermaphroditus* (Viverridae), as a seed disperser for large-seeded plants. Mammal. Study.

[63] Soulé ME (1988). Reconstructed dynamics of rapid extinctions of chaparral‐requiring birds in urban habitat islands. Conserv. Biol..

[64] Crooks KR, Soulé ME (1999). Mesopredator release and avifaunal extinctions in a fragmented system. Nature.

[65] Brashares, J. S., Prugh, L. R., Stoner, C. J. & Epps, C. W. in *Trophic cascades: predators, prey, and the changing dynamics of nature*, 221–240.(2010).

[66] McKinney ML, Lockwood JL (1999). Biotic homogenization: a few winners replacing many losers in the next mass extinction. Trends Ecol. Evolution.

[67] Rodríguez JP, Brotons L, Bustamante J, Seoane J (2007). The application of predictive modelling of species distribution to biodiversity conservation. Diversity Distrib..

[68] Guisan A (2013). Predicting species distributions for conservation decisions. Ecol. Lett..

[69] Elith J, Leathwick JR (2009). Species distribution models: ecological explanation and prediction across space and time. Annu. Rev. Ecol., Evolution, Syst..

[70] Lippitt CD (2008). Incorporating anthropogenic variables into a species distribution model to map gypsy moth risk. Ecol. Model..

[71] Bush A (2017). Connecting Earth observation to high-throughput biodiversity data. Nat. Ecol. Evolution.

[72] Tilker, A. et al. Identifying conservation priorities in a defaunated tropical biodiversity hotspot. BioRxiv [Preprint] available from: 10.1101/790766 (2019).

[73] Babweteera F, Brown N (2009). Can remnant frugivore species effectively disperse tree seeds in secondary tropical rain forests?. Biodivers. Conserv..

[74] Gerland P (2014). World population stabilization unlikely this century. Science.

[75] Meijer JR, Huijbregts MA, Schotten KC, Schipper AM (2018). Global patterns of current and future road infrastructure. Environ. Res. Lett..

[76] Lagan P, Mannan S, Matsubayashi H (2007). Sustainable use of tropical forests by reduced-impact logging in Deramakot Forest Reserve, Sabah, Malaysia. Sustainability and Diversity of Forest Ecosystems.

[77] Abrams JF (2018). Studying terrestrial mammals in tropical rainforests. A user guide for camera-trapping and environmental DNA..

[78] Asner GP, Keller M, Pereira R, Zweede JC, Silva JN (2004). Canopy damage and recovery after selective logging in Amazonia: field and satellite studies. Ecol. Appl..

[79] GIMP team, GNU Image Manipulation Program. https://www.gimp.org. (2017)

[80] R Core Team R: A language and environment for statistical computing. R Foundation for Statistical Computing, Vienna, Austria. https://www.R-project.org/. (2018).

[81] Muchaal PK, Ngandjui G (1999). Impact of village hunting on wildlife populations in the western Dja Reserve, Cameroon. Conserv. Biol..

[82] Abrahams MI, Peres CA, Costa HC (2017). Measuring local depletion of terrestrial game vertebrates by central-place hunters in rural Amazonia. PloS One.

[83] Koerner SE, Poulsen JR, Blanchard EJ, Okouyi J, Clark CJ (2017). Vertebrate community composition and diversity declines along a defaunation gradient radiating from rural villages in Gabon. J. Appl. Ecol..

[84] QGIS Development Team QGIS Geographic Information System. Open Source Geospatial Foundation Project. http://qgis.osgeo.org. (2019).

[85] Hijmans, R. J. et al. Package ‘raster’. R package.(2015).

[86] Abramov AV, Duckworth JW, Wang YX, Roberton SI (2008). The stripe‐backed weasel Mustela strigidorsa: taxonomy, ecology, distribution and status. Mammal. Rev..

[87] Supparatvikorn, S. et al. (2012). Discovery of the Yellow-bellied Weasel *Mustela Kathiah* In Thailand. Natural History Bulletin of the Siam Society, 58.

[88] Ross J, Hearn AJ, Macdonald DW (2013). Recent camera-trap records of Malay weasel *Mustela nudipes* in Sabah, Malaysian Borneo. Small Carniv. Conserv..

[89] Timmins, R. & Duckworth, J. W. *Muntiacus rooseveltorum*. The IUCN Red List of Threatened Species 2016: e.T13928A22160435. (2016).

[90] Timmins, R. & Duckworth, J. W. *Muntiacus truongsonensis*. The IUCN Red List of Threatened Species 2016: e.T44704A22154056. (2016).

[91] Francis, C. *Field guide to the mammals of South-east Asia*. (Bloomsbury Publishing, 2019)

[92] MacKenzie, D. I. et al. *Occupancy estimation and modeling: inferring patterns and dynamics of species occurrence*. (Elsevier, 2017)

[93] Plummer, M. rjags: Bayesian Graphical Models using MCMC. R package version 4–8. https://CRAN.R-project.org/package=rjags (2018)

[94] Gelman A, Carlin JB, Stern HS, Rubin DB (2004). Bayesian Data Analysis..

