# Supplementary material for: Habitat degradation and indiscriminate hunting differentially impact faunal communities in the Southeast Asian tropical biodiversity hotspot
Source: Commun Biol. 2019 Oct 30;2:396. doi: 10.1038/s42003-019-0640-y (PMC6821809; doi:10.1038/s42003-019-0640-y)
Supplement: Supplementary file 1 — Supplementary information [file 42003_2019_640_MOESM1_ESM.pdf]

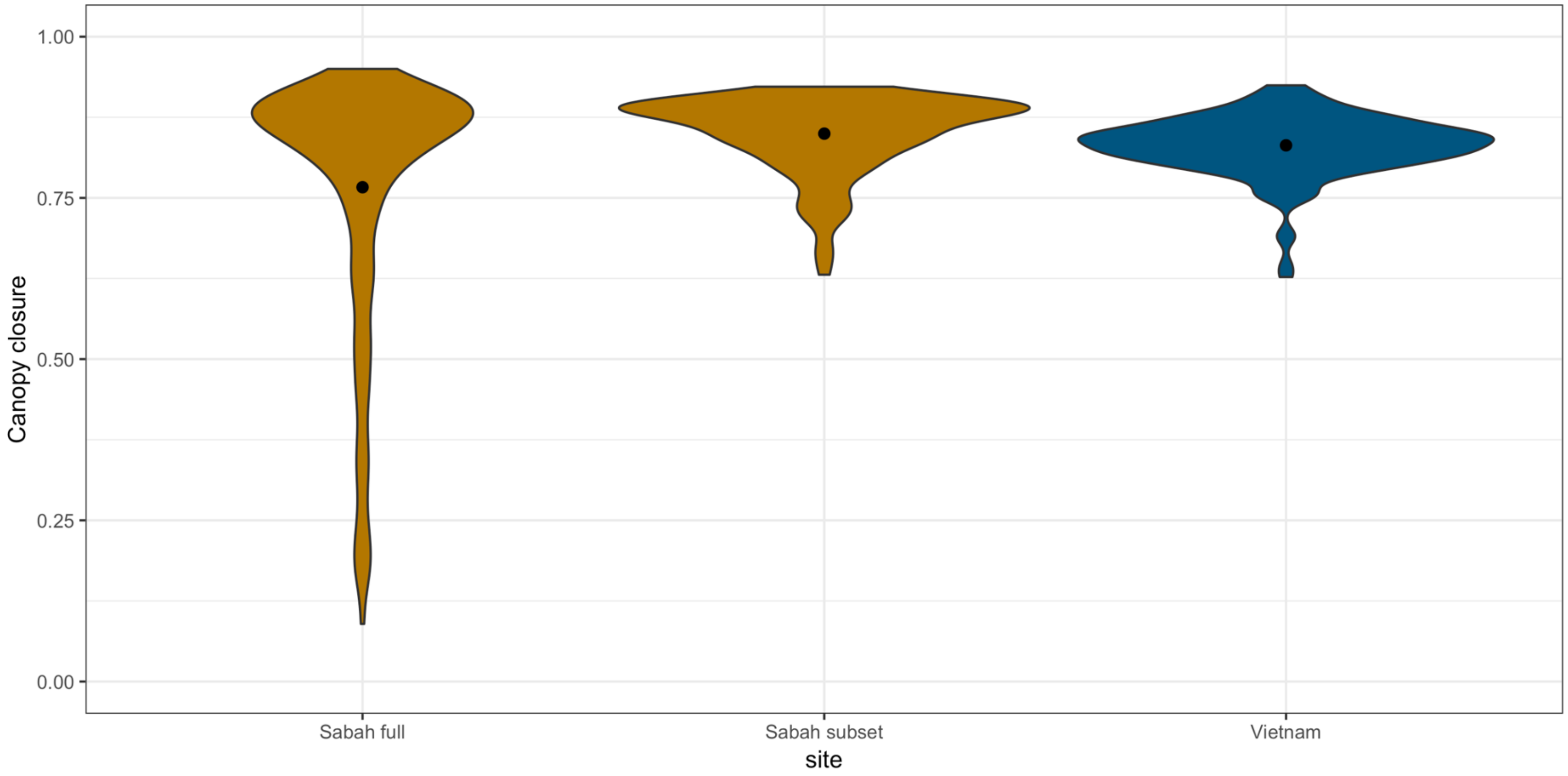

Supplementary Figure 1: Violin plots for the canopy closure values used in the subsetted Bayesian occupancy model. Left: Full range of canopy closure values from all sampling stations in the degraded sites. Middle: Subset of canopy closure values with mean and variation similar to full range of canopy closure values for the hunted sites. Right: Full range of canopy closure values for the hunted sites. Black dot shows mean. The model with the subsetted canopy closure values for the degraded sites was run to determine if the species responses in the degraded sites was primarily driven by the greater variation in canopy closure at the degraded sites.

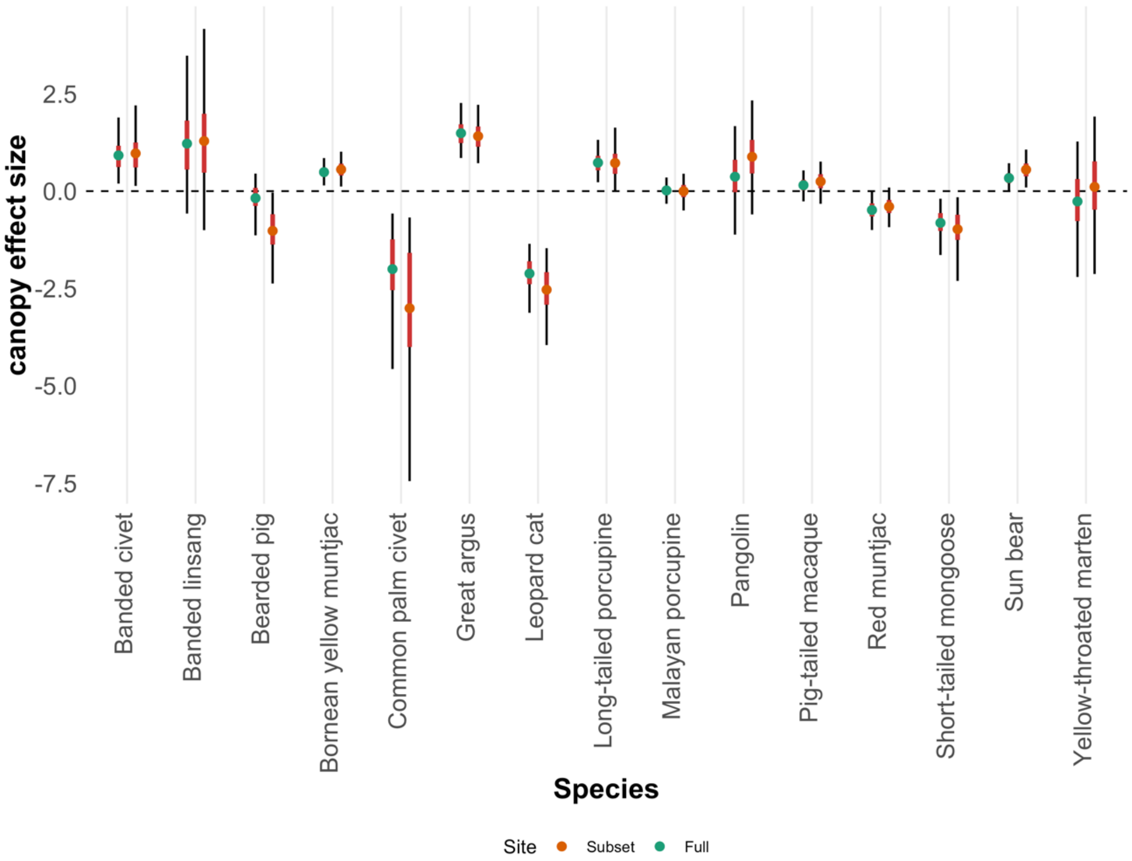

Supplementary Figure 2: Mean effect sizes for canopy closure for the 15 species from Malaysian Borneo used in the Bayesian community occupancy model. Effect sizes from the full canopy closure dataset for the degraded sites are shown in green, effect sizes from the subsetted canpopy closure dataset for the degraded sites are shown in orange (see SI Fig. 2). Both the full and subset models show similar results.

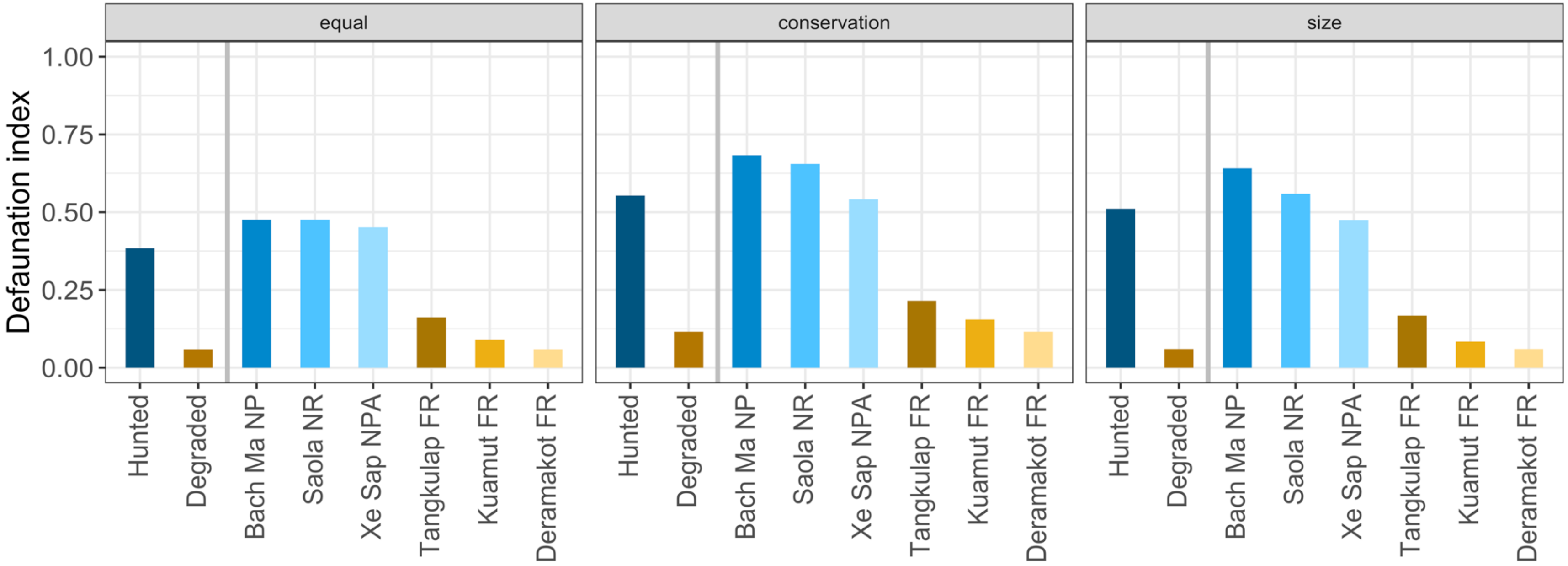

Supplementary Figure 3: Historical defaunation indexes for hunted and degraded. Defaunation values were calculated using a measure of functional extinction, defined as species recorded in < 2.5% of camera trap stations per site. Larger and more threatened species have higher levels of functional extinction. Species importance is weighted in three ways: equally where all species are considered equally important (equal), based on conservation status (conservation), and based on species average body size using a ranked species order (size).

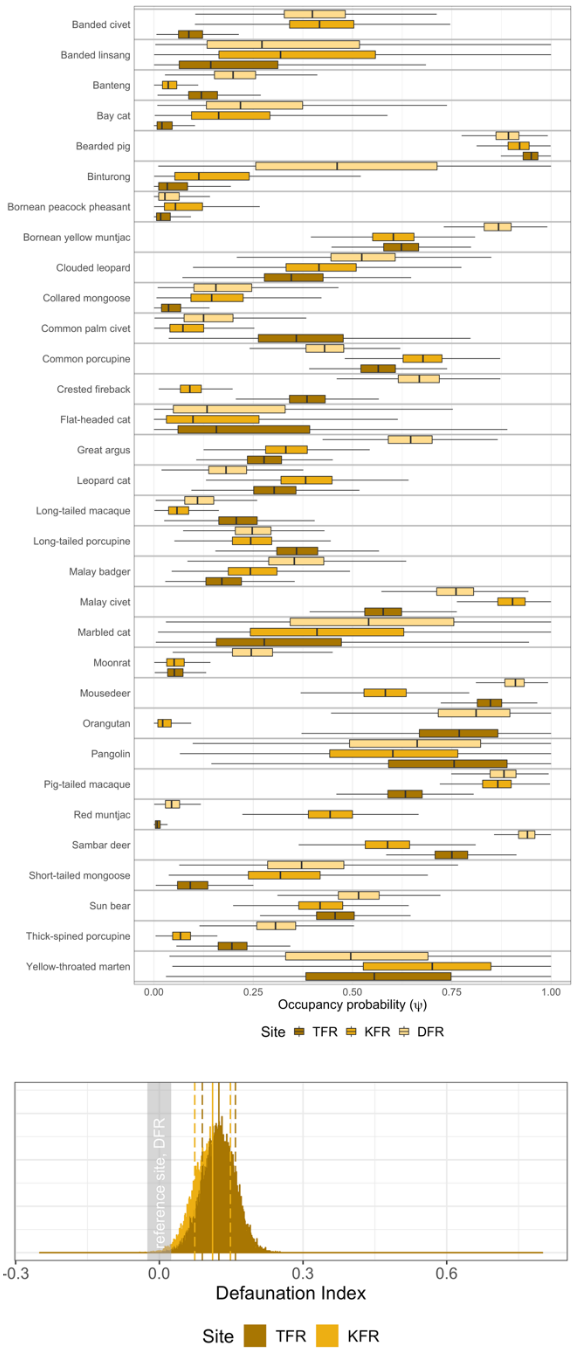

Supplementary Figure 4: (A) Bayesian community occupancy estimates for all mammal and ground-dwelling bird species in Malaysian Borneo. Boxplots represent occupancy estimates (mean and 95% BCI) for that species at that site. **(B)** Defaunation index values for all species for Tangkulap FR and Kuamut FR, using Deramakot FR (least degraded) as the reference site.

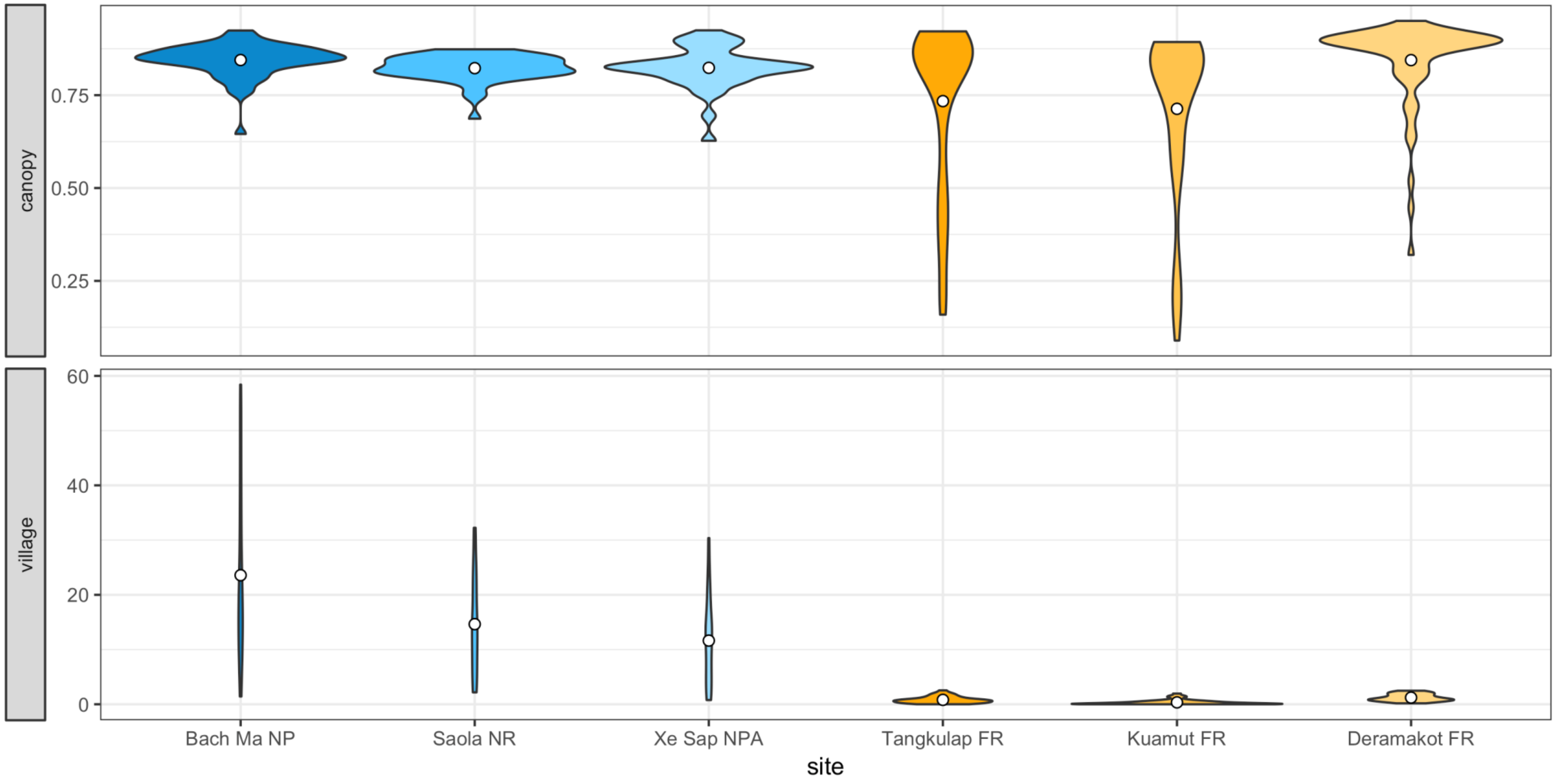

Supplementary Figure 5: Violin plots for two covariates, canopy closure and village density, used in the Bayesian community occupancy analyses. White circle denotes mean value for that covariate and site.

Supplementary Table 1: Species used in the historical defaunation index for the hunted and degraded sites. The historical reference assemblage, defined as species that occurred historically at the sites, is comprised by all species in the table. The current reference assemblage, defined as species recorded during the surveys with a naïve occupancy of > 2.5% of the total camera trap stations, is denoted by "1" in the present / absent column. If a species historically occurred at the sites but was not recorded in our surveys, or the species was recorded but at < 2.5% of the total camera-trap stations, the species was defined as functionally extinct, and given a value of "0" in the present / absent column. Original values for species *IUCN Red List* status and average body size are provided, as well as the converted weights.

Bach Ma NP (hunted / Vietnam)						
**Common name**	**Scientific name**	**Present / absent**	**IUCN Red List**	**Average body size**	**Conservation w_k**	**Size w_k**
Annamite dark muntjac	*Muntiacus rooseveltorum / truongsonensis*	1	DD	28	2.5	12.17
Annamite striped rabbit	*Nesolagus timminsi*	1	DD	2	2.5	1.68
Asian elephant	*Elephas maximus*	0	EN	2700	4	374.56
Asiatic black bear	*Ursus thibetanus*	0	VU	115	3	35.12
Asiatic brush-tailed porcupine	*Atherurus macrourus*	1	LC	2	1	1.68
Asiatic golden cat	*Catopuma temminckii*	0	NT	18	2	8.74
Binturong	*Arctictis binturong*	0	VU	20	3	9.46
Common palm civet	*Paradoxurus hermaphroditus*	1	LC	3.2	1	2.39
Crab-eating mongoose	*Herpestes urva*	1	LC	2.5	1	1.99
Crested argus	*Rheinardia ocellata*	0	EN	2	4	1.68
Edwards's pheasant	*Lophura edwardsi*	0	CR	2	5	1.68
Eurasian wild pig	*Sus scrofa*	1	LC	45	1	17.37
Ferret badger	*Melogale* spp.	1	LC	1.3	1	1.22
Gaur	*Bos gaurus*	0	VU	615	3	123.50
Gray peacock pheasant	*Polyplectron bicalcaratum*	0	LC	2.5	1	1.99
Green peafowl	*Pavo muticus*	0	EN	2.5	4	1.99
Javan rhinocerous	*Rhinoceros sondaicus*	0	CR	1500	5	241.03

Large Indian civet	*Viverra zibetha*	0	LC	5	4	3.34
Large spotted civet	*Viverra megaspila*	0	EN	8	1	4.76
Large-antlered muntjac	*Muntiacus vuquangensis*	0	CR	60	5	21.56
Leopard	*Panthera pardus*	0	VU	45	3	17.37
Leopard cat	*Prionailurus bengalensis*	1	LC	4	1	2.83
Lesser mouse deer	*Tragulus kanchil*	0	LC	2	1	1.68
Mainland clouded leopard	*Neofelis nebulosa*	0	VU	15	3	7.62
Malayan porcupine	*Hystrix brachyura*	1	LC	2.2	1	1.81
Marbled cat	*Pardofelis marmorata*	0	NT	2.2	2	1.81
Masked palm civet	*Paguma larvata*	1	LC	3.2	1	2.39
Northern pig-tailed macaque	*Macaca leonina*	1	LC	10	1	5.62
Northern red muntjac	*Muntiacus vaginalis*	0	LC	90	1	29.22
Owston's civet	*Chrotogale owstoni*	0	EN	6	4	3.83
Pangolin	*Manis* spp.	0	CR	4.5	5	3.09
Red junglefowl	*Gallus gallus*	1	LC	2	1	1.68
Rhesus macaque	*Macaca mulatta*	0	LC	10	1	5.62
Sambar	*Rusa unicolor*	0	VU	82	3	27.25
Saola	*Pseudoryx nghetinhensis*	0	CR	90	5	29.22
Serow	*Capricornis milneedwardsii*	0	NT	70	2	24.20
Siamese fireback	*Lophura diardi*	0	LC	2	1	1.68
Silver pheasant	*Lophura nycthemera*	1	LC	2	1	1.99
Small Asian mongoose	*Herpestes javanicus*	0	LC	1.5	1	1.36
Small Indian civet	*Viverricula indica*	0	LC	1.5	1	1.36
Spotted linsang	*Prionodon pardicolor*	1	LC	2	1	1.68
Stump-tailed macaque	*Macaca arctoides*	1	LC	10	1	5.62
Sun bear	*Helarctos malayanus*	0	VU	55	3	20.20
Tiger	*Panthera tigris*	0	EN	135	4	39.61
Yellow-throated marten	*Martes flavigula*	1	LC	2.5	1	1.99

**Saola Nature Reserves (hunted / Vietnam)**						
**Common name**	**Scientific name**	**Present / absent**	**IUCN Red List**	**Average body size**	**Conservation w_k**	**Size w_k**
Annamite dark muntjac	*Muntiacus rooseveltorum / truongsonensis*	1	DD	28	2.5	12.17
Annamite striped rabbit	*Nesolagus timminsi*	1	DD	2	2.5	1.68

Asian elephant	*Elephas maximus*	0	EN	2700	4	374.56
Asiatic black bear	*Ursus thibetanus*	0	VU	115	3	35.12
Asiatic brush-tailed porcupine	*Atherurus macrourus*	1	LC	2	1	1.68
Asiatic golden cat	*Catopuma temminckii*	0	NT	18	2	8.74
Binturong	*Arctictis binturong*	0	VU	20	3	9.46
Common palm civet	*Paradoxurus hermaphroditus*	1	LC	3.2	1	2.39
Crab-eating mongoose	*Herpestes urva*	1	LC	2.5	1	1.99
Crested argus	*Rheinardia ocellata*	1	EN	2	4	1.68
Edwards's pheasant	*Lophura edwardsi*	0	CR	2	5	1.68
Eurasian wild pig	*Sus scrofa*	1	LC	45	1	17.37
Ferret badger	*Melogale* spp.	1	LC	1.3	1	1.22
Gaur	*Bos gaurus*	0	VU	615	3	123.50
Gray peacock pheasant	*Polyplectron bicalcaratum*	0	LC	2.5	1	1.99
Green peafowl	*Pavo muticus*	0	EN	2.5	4	1.99
Javan rhinocerous	*Rhinoceros sondaicus*	0	CR	1500	5	241.03
Large Indian civet	*Viverra zibetha*	0	LC	5	4	3.34
Large spotted civet	*Viverra megaspila*	0	EN	8	1	4.76
Large-antlered muntjac	*Muntiacus vuquangensis*	0	CR	60	5	21.56
Leopard	*Panthera pardus*	0	VU	45	3	17.37
Leopard cat	*Prionailurus bengalensis*	1	LC	4	1	2.83
Lesser mouse deer	*Tragulus kanchil*	0	LC	2	1	1.68
Mainland clouded leopard	*Neofelis nebulosa*	0	VU	15	3	7.62
Malayan porcupine	*Hystrix brachyura*	1	LC	2.2	1	1.81
Marbled cat	*Pardofelis marmorata*	0	NT	2.2	2	1.81
Masked palm civet	*Paguma larvata*	1	LC	3.2	1	2.39
Northern pig-tailed macaque	*Macaca leonina*	1	LC	10	1	5.62
Northern red muntjac	*Muntiacus vaginalis*	1	LC	90	1	29.22
Owston's civet	*Chrotogale owstoni*	0	EN	6	4	3.83
Pangolin	*Manis* spp.	0	CR	4.5	5	3.09
Red junglefowl	*Gallus gallus*	0	LC	2	1	1.68
Rhesus macaque	*Macaca mulatta*	0	LC	10	1	5.62
Sambar	*Rusa unicolor*	0	VU	82	3	27.25
Saola	*Pseudoryx nghetinhensis*	0	CR	90	5	29.22

Serow	*Capricornis milneedwardsii*	1	NT	70	2	24.20
Siamese fireback	*Lophura diardi*	0	LC	2	1	1.68
Silver pheasant	*Lophura nycthemera*	0	LC	2	1	1.99
Small Asian mongoose	*Herpestes javanicus*	0	LC	1.5	1	1.36
Small Indian civet	*Viverricula indica*	0	LC	1.5	1	1.36
Spotted linsang	*Prionodon pardicolor*	1	LC	2	1	1.68
Stump-tailed macaque	*Macaca arctoides*	1	LC	10	1	5.62
Sun bear	*Helarctos malayanus*	0	VU	55	3	20.20
Tiger	*Panthera tigris*	0	EN	135	4	39.61
Yellow-throated marten	*Martes flavigula*	0	LC	2.5	1	1.99

**Xe Sap / Palé (hunted / Laos)**						
**Common name**	**Scientific name**	**Present / absent**	**IUCN Red List**	**Average body size**	**Conservation w_k**	**Size w_k**
Annamite dark muntjac	*Muntiacus rooseveltorum / truongsonensis*	1	DD	28	2.5	12.17
Annamite striped rabbit	*Nesolagus timminsi*	1	DD	2	2.5	1.68
Asian elephant	*Elephas maximus*	0	EN	2700	4	374.56
Asiatic black bear	*Ursus thibetanus*	1	VU	115	3	35.12
Asiatic brush-tailed porcupine	*Atherurus macrourus*	0	LC	2	1	1.68
Asiatic golden cat	*Catopuma temminckii*	0	NT	18	2	8.74
Binturong	*Arctictis binturong*	0	VU	20	3	9.46
Common palm civet	*Paradoxurus hermaphroditus*	1	LC	3.2	1	2.39
Crab-eating mongoose	*Herpestes urva*	1	LC	2.5	1	1.99
Crested argus	*Rheinardia ocellata*	0	EN	2	4	1.68
Edwards's pheasant	*Lophura edwardsi*	0	CR	2	5	1.68
Eurasian wild pig	*Sus scrofa*	1	LC	45	1	17.37
Ferret badger	*Melogale spp.*	1	LC	1.3	1	1.22
Gaur	*Bos gaurus*	0	VU	615	3	123.50
Gray peacock pheasant	*Polyplectron bicalcaratum*	0	LC	2.5	1	1.99
Green peafowl	*Pavo muticus*	0	EN	2.5	4	1.99
Javan rhinocerous	*Rhinoceros sondaicus*	0	CR	1500	5	241.03
Large Indian civet	*Viverra zibetha*	0	LC	5	4	3.34
Large spotted civet	*Viverra megaspila*	0	EN	8	1	4.76

Large-antlered muntjac	*Muntiacus vuquangensis*	0	CR	60	5	21.56
Leopard	*Panthera pardus*	0	VU	45	3	17.37
Leopard cat	*Prionailurus bengalensis*	1	LC	4	1	2.83
Lesser mouse deer	*Tragulus kanchil*	0	LC	2	1	1.68
Mainland clouded leopard	*Neofelis nebulosa*	0	VU	15	3	7.62
Malayan porcupine	*Hystrix brachyura*	0	LC	2.2	1	1.81
Marbled cat	*Pardofelis marmorata*	0	NT	2.2	2	1.81
Masked palm civet	*Paguma larvata*	1	LC	3.2	1	2.39
Northern pig-tailed macaque	*Macaca leonina*	0	LC	10	1	5.62
Northern red muntjac	*Muntiacus vaginalis*	1	LC	90	1	29.22
Owston's civet	*Chrotogale owstoni*	1	EN	6	4	3.83
Pangolin	*Manis spp.*	1	CR	4.5	5	3.09
Red junglefowl	*Gallus gallus*	0	LC	2	1	1.68
Rhesus macaque	*Macaca mulatta*	1	LC	10	1	5.62
Sambar	*Rusa unicolor*	0	VU	82	3	27.25
Saola	*Pseudoryx nghetinhensis*	0	CR	90	5	29.22
Serow	*Capricornis milneedwardsii*	1	NT	70	2	24.20
Siamese fireback	*Lophura diardi*	0	LC	2	1	1.68
Silver pheasant	*Lophura nycthemera*	0	LC	2	1	1.99
Small Asian mongoose	*Herpestes javanicus*	0	LC	1.5	1	1.36
Small Indian civet	*Viverricula indica*	0	LC	1.5	1	1.36
Spotted linsang	*Prionodon pardicolor*	1	LC	2	1	1.68
Stump-tailed macaque	*Macaca arctoides*	1	LC	10	1	5.62
Sun bear	*Helarctos malayanus*	0	VU	55	3	20.20
Tiger	*Panthera tigris*	0	EN	135	4	39.61
Yellow-throated marten	*Martes flavigula*	1	LC	2.5	1	1.99

**Deramakot FR (degraded / Malaysian Borneo)**						
**Common name**	**Scientific name**	**Present / absent**	**IUCN Red List**	**Average body size**	**Conservation w_k**	**Size w_k**
Asian elephant	*Elephas maximus*	1	EN	2000	4	299.07
Banded civet	*Hemigalus derbyanus*	1	NT	6	2	3.83
Banded linsang	*Prionodon linsang*	1	LC	2	1	1.68
Banteng	*Bos javanicus*	1	EN	545	4	112.80

Bay cat	*Pardofelis badia*	1	EN	3.4	4	2.50
Bearded pig	*Sus barbatus*	1	VU	100	3	31.62
Binturong	*Arctictis binturong*	1	VU	20	3	9.46
Bornean ground cuckoo	*Carpococcyx radiceus*	1	NT	1	2	1.00
Bornean peacock pheasant	*Polyplectron schleiermacheri*	0	EN	2.5	4	1.99
Bornean yellow muntjac	*Muntiacus atherodes*	1	NT	28	2	12.17
Collared mongoose	*Herpestes semitorquatus*	1	NT	2.5	1	1.99
Common palm civet	*Paradoxurus hermaphroditus*	1	LC	3.2	1	2.39
Crested fireback	*Lophura ignita*	1	NT	2.5	2	1.99
Flat-headed cat	*Prionailurus planiceps*	0	EN	2	4	1.68
Greater argus	*Argusianus argus*	1	NT	2.5	2	1.99
Leopard cat	*Prionailurus bengalensis*	1	LC	4	1	2.83
Long-tailed macaque	*Macaca fascicularis*	1	LC	5	1	3.34
Long-tailed porcupine	*Trichys fasciculata*	1	LC	2	1	1.68
Malay civet	*Viverra tangalunga*	1	LC	4.5	1	3.09
Malay porcupine	*Hystrix brachyura*	1	LC	2.2	1	1.81
Marbled cat	*Pardofelis marmorata*	1	NT	2.2	2	1.81
Moonrat	*Echinosorex gymnura*	1	LC	0.45	1	0.55
Mouse deer	*Tragulus* spp.	1	LC	2	1	1.68
Orangutan	*Pongo pygmaeus*	1	CR	58	5	21.02
Otter civet	*Cynogale bennettii*	0	EN	3.8	4	2.72
Pangolin	*Manis* spp.	1	CR	4.5	5	3.09
Sambar	*Rusa unicolor*	1	VU	82	3	27.25
Short-tailed mongoose	*Herpestes brachyurus*	1	NT	1.5	1	1.36
Southern pig-tailed macaque	*Macaca nemestrina*	1	LC	10	1	5.62
Southern red muntjac	*Muntiacus muntjak*	1	LC	90	1	29.22
Sumatran rhinocerous	*Dicerorhinus sumatrensis*	0	CR	700	5	136.09
Sun bear	*Helarctos malayanus*	1	VU	55	3	20.20
Sunda clouded leopard	*Neofelis diardi*	1	VU	35	3	14.39
Sunda stink badger	*Mydaus javanensis*	1	LC	2.2	1	1.81
Thick-spined porcupine	*Hystrix crassispinis*	1	LC	1.8	1	1.55
Yellow-throated marten	*Martes flavigula*	1	LC	2.5	1	1.99

Tangkulap FR (degraded / Malaysian Borneo)						
**Common name**	**Scientific name**	**Present / absent**	**IUCN Red List**	**Average body size**	**Conservation w_k**	**Size w_k**
Asian elephant	*Elephas maximus*	1	EN	2000	4	299.07
Banded civet	*Hemigalus derbyanus*	0	NT	6	2	3.83
Banded linsang	*Prionodon linsang*	0	LC	2	1	1.68
Banteng	*Bos javanicus*	1	EN	545	4	112.80
Bay cat	*Pardofelis badia*	0	EN	3.4	4	2.50
Bearded pig	*Sus barbatus*	1	VU	100	3	31.62
Binturong	*Arctictis binturong*	0	VU	20	3	9.46
Bornean ground cuckoo	*Carpococcyx radiceus*	1	NT	1	2	1.00
Bornean peacock pheasant	*Polyplectron schleiermacheri*	0	EN	2.5	4	1.99
Bornean yellow muntjac	*Muntiacus atherodes*	1	NT	28	2	12.17
Collared mongoose	*Herpestes semitorquatus*	0	NT	2.5	1	1.99
Common palm civet	*Paradoxurus hermaphroditus*	1	LC	3.2	1	2.39
Crested fireback	*Lophura ignita*	1	NT	2.5	2	1.99
Flat-headed cat	*Prionailurus planiceps*	0	EN	2	4	1.68
Greater argus	*Argusianus argus*	1	NT	2.5	2	1.99
Leopard cat	*Prionailurus bengalensis*	1	LC	4	1	2.83
Long-tailed macaque	*Macaca fascicularis*	1	LC	5	1	3.34
Long-tailed porcupine	*Trichys fasciculata*	1	LC	2	1	1.68
Malay civet	*Viverra tangalunga*	1	LC	4.5	1	3.09
Malay porcupine	*Hystrix brachyura*	1	LC	2.2	1	1.81
Marbled cat	*Pardofelis marmorata*	1	NT	2.2	2	1.81
Moonrat	*Echinosorex gymnura*	1	LC	0.45	1	0.55
Mouse deer	*Tragulus* spp.	1	LC	2	1	1.68
Orangutan	*Pongo pygmaeus*	1	CR	58	5	21.02
Otter civet	*Cynogale bennettii*	0	EN	3.8	4	2.72
Pangolin	*Manis* spp.	1	CR	4.5	5	3.09
Sambar	*Rusa unicolor*	1	VU	82	3	27.25
Short-tailed mongoose	*Herpestes brachyurus*	1	NT	1.5	1	1.36
Southern pig-tailed macaque	*Macaca nemestrina*	1	LC	10	1	5.62
Southern red muntjac	*Muntiacus muntjak*	0	LC	90	1	29.22

Sumatran rhinocerous	*Dicerorhinus sumatrensis*	0	CR	700	5	136.09
Sun bear	*Helarctos malayanus*	1	VU	55	3	20.20
Sunda clouded leopard	*Neofelis diardi*	1	VU	35	3	14.39
Sunda stink badger	*Mydaus javanensis*	1	LC	2.2	1	1.81
Thick-spined porcupine	*Hystrix crassispinis*	1	LC	1.8	1	1.55
Yellow-throated marten	*Martes flavigula*	1	LC	2.5	1	1.99

**Kuamut FR (degraded / Malaysian Borneo)**						
**Common name**	**Scientific name**	**Present / absent**	**IUCN Red List**	**Average body size**	**Conservation w_k**	**Size w_k**
Asian elephant	*Elephas maximus*	1	EN	2000	4	299.07
Banded civet	*Hemigalus derbyanus*	1	NT	6	2	3.83
Banded linsang	*Prionodon linsang*	1	LC	2	1	1.68
Banteng	*Bos javanicus*	1	EN	545	4	112.80
Bay cat	*Pardofelis badia*	1	EN	3.4	4	2.50
Bearded pig	*Sus barbatus*	1	VU	100	3	31.62
Binturong	*Arctictis binturong*	0	VU	20	3	9.46
Bornean ground cuckoo	*Carpococcyx radiceus*	0	NT	1	2	1.00
Bornean peacock pheasant	*Polyplectron schleiermacheri*	0	EN	2.5	4	1.99
Bornean yellow muntjac	*Muntiacus atherodes*	1	NT	28	2	12.17
Collared mongoose	*Herpestes semitorquatus*	1	NT	2.5	1	1.99
Common palm civet	*Paradoxurus hermaphroditus*	1	LC	3.2	1	2.39
Crested fireback	*Lophura ignita*	1	NT	2.5	2	1.99
Flat-headed cat	*Prionailurus planiceps*	0	EN	2	4	1.68
Greater argus	*Argusianus argus*	1	NT	2.5	2	1.99
Leopard cat	*Prionailurus bengalensis*	1	LC	4	1	2.83
Long-tailed macaque	*Macaca fascicularis*	1	LC	5	1	3.34
Long-tailed porcupine	*Trichys fasciculata*	1	LC	2	1	1.68
Malay civet	*Viverra tangalunga*	1	LC	4.5	1	3.09
Malay porcupine	*Hystrix brachyura*	1	LC	2.2	1	1.81
Marbled cat	*Pardofelis marmorata*	1	NT	2.2	2	1.81
Moonrat	*Echinosorex gymnura*	1	LC	0.45	1	0.55
Mouse deer	*Tragulus* spp.	1	LC	2	1	1.68
Orangutan	*Pongo pygmaeus*	1	CR	58	5	21.02

Otter civet	*Cynogale bennettii*	0	EN	3.8	4	2.72
Pangolin	*Manis* spp.	1	CR	4.5	5	3.09
Sambar	*Rusa unicolor*	1	VU	82	3	27.25
Short-tailed mongoose	*Herpestes brachyurus*	1	NT	1.5	1	1.36
Southern pig-tailed macaque	*Macaca nemestrina*	1	LC	10	1	5.62
Southern red muntjac	*Muntiacus muntjak*	1	LC	90	1	29.22
Sumatran rhinocerous	*Dicerorhinus sumatrensis*	0	CR	700	5	136.09
Sun bear	*Helarctos malayanus*	1	VU	55	3	20.20
Sunda clouded leopard	*Neofelis diardi*	1	VU	35	3	14.39
Sunda stink badger	*Mydaus javanensis*	1	LC	2.2	1	1.81
Thick-spined porcupine	*Hystrix crassispinis*	1	LC	1.8	1	1.55
Yellow-throated marten	*Martes flavigula*	1	LC	2.5	1	1.99

Supplementary Table 2: Species and sister species pairs for hunted (Vietnam / Laos) and degraded (Malaysian Borneo) landscapes used in the comparative Bayesian community occupancy analysis. Original values for species IUCN Red List status and average body size are provided, as well as the converted weights (w_k).

No.	Species Vietnam / Laos	Species Malaysian Borneo	Average body size (kg)	Feeding ecology (*1-3*)
1	Dark muntjac *Muntiacus rooseveltorum / truongsonensis*	Bornean yellow muntjac *Muntiacus atherodes*	20 / 20	Forest browsers, known to feed on variety of trees, shrubs, and forbs.
2	Asian black bear *Ursus thibetanus*	Sun bear *Helarctos malayanus*	100 / 60	Omnivorous, highly generalist. Known to feed on a wide range of plant and animal matter matter.
3	Asiatic brush tailed porcupine *Atherurus macrourus*	Long-tailed porcupine *Trichys fasciculata*	2 / 2	Generalist herbivore, consumes wide range of plant material, including leaves, roots, fruit. Has been known to feed on insects and small vertebrates.
4	Common palm civet *Paradoxurus hermaphroditus*	Common palm civet *Paradoxurus hermaphroditus*	3 / 3	Omnivorous, highly generalist. Feeds on berries and fruits, as well as small mammals and insects.
5	Crab-eating mongoose *Herpestes urva*	Short-tailed mongoose *Herpestes brachyurus*	3 / 3	Carnivorous, highly generalist. Feeds on variety of invertebrates and small vertebrates.
6	Crested argus *Rheinardia ocellata*	Great argus *Argusianus argus*	2 / 3	Generalist omnivore, feeds on fruit, seeds, flowers, leaf buds, and invertebrates.
7	Eurasian wild pig *Sus scrofa*	Bearded pig *Sus barbatus*	60 / 80	Generalist omnivore, highly versatile feeding ecology. Known to feed on wide range of plant and animal matter. Sometimes scavenges.
8	Leopard cat *Prionailurus bengalensis*	Leopard cat *Prionailurus bengalensis*	4 / 4	Generalist carnivore, feeds on variety of small mammals and invertebrates.
9	Malayan porcupine *Hystrix brachyura*	Malay porcupine *Hystrix brachyura*	2 / 2	Generalist herbivore, consumes wide range of plant material, including leaves, roots, fruit. Has been known to feed on insects and small vertebrates.
10	Northern pig-tailed macaque *Macaca*	Southern pig-tailed macaque *Macaca*	10 / 10	Highly versatile omnivore, known to feed on fruits, seeds,

	leonina	*nemestrina*		invertebrates, and small vertebrates.
11	Red muntjac *Muntiacus vaginalis*	Southern red muntjac *Muntiacus muntjak*	30 / 30	Forest browsers, known to feed on variety of trees, shrubs, and forbs.
12	Owston's civet *Chrotogale owstoni*	Banded civet *Hemigalus derbyanus*	6 / 6	Carnivorous, feeds on invertebrates and small vertebrates.
13	Pangolin *Manis* spp.	Sunda pangolin *Manis javanica*	5 / 5	Insectivorous, particularly termites and ants.
14	Spotted linsang *Prionodon pardicolor*	Banded linsang *Prionodon linsang*	2 / 2	Carnivorous, feeds on invertebrates and small vertebrates.
15	Yellow-throated marten *Martes flavigula*	Yellow-throated marten *Martes flavigula*	3 / 3	Carnivorous, feeds on invertebrates and small vertebrates.

Supplementary Table 3: Bayesian occupancy summary statistics for the 15 species and sister species pairs for hunted (Vietnam / Laos) and degraded (Malaysian Borneo) sites used in the community occupancy analysis. Table shows mean, standard deviation, and the 2.5% and 97.5% values for the posterior occupancy estimates.

**Bach Ma NP (hunted / Vietnam)**						
**No.**	**Common name**	**Scientific name**	**Mean**	**SD**	**2.50%**	**97.50%**
1	Dark muntjac	*Muntiacus rooseveltorum / truongsonensis*	0.1541	0.0968	0.0331	0.4076
2	Asian black bear	*Ursus thibetanus*	0.1597	0.1714	0.0029	0.6536
3	Asiatic brush tailed porcupine	*Atherurus macrourus*	0.4454	0.0813	0.2947	0.6127
4	Common palm civet	*Paradoxurus hermaphroditus*	0.4437	0.1020	0.2658	0.6644
5	Crab eating mongoose	*Herpestes urva*	0.2710	0.0745	0.1417	0.4339
6	Crested argus	*Rheinardia ocellata*	0.0640	0.0558	0.0058	0.2089
7	Eurasian wild pig	*Sus scrofa*	0.6144	0.0906	0.4383	0.7927
8	Leopard cat	*Prionailurus bengalensis*	0.5090	0.1545	0.2503	0.8474
9	Malayan porcupine	*Hystrix brachyura*	0.2212	0.1376	0.0482	0.5851
10	Northern pig-tailed macaque	*Macaca leonina*	0.4032	0.0936	0.2370	0.6012
11	Red muntjac	*Muntiacus vaginalis*	0.0718	0.0421	0.0146	0.1752
12	Owston's civet	*Chrotogale owstoni*	0.1601	0.1703	0.0028	0.6428
13	Pangolin	*Manis* spp.	0.1135	0.1258	0.0023	0.4734
14	Spotted linsang	*Prionodon pardicolor*	0.3613	0.1777	0.1141	0.8156
15	Yellow-throated marten	*Martes flavigula*	0.2685	0.1465	0.0799	0.6502

**Hue and Quang Nam SNRs (hunted / Vietnam)**						
**No.**	**Common name**	**Scientific name**	**Mean**	**SD**	**2.50%**	**97.50%**
1	Dark muntjac	*Muntiacus rooseveltorum / truongsonensis*	0.2868	0.1295	0.0915	0.5894

2	Asian black bear	*Ursus thibetanus*	0.2488	0.1660	0.0131	0.6234
3	Asiatic brush tailed porcupine	*Atherurus macrourus*	0.2904	0.0682	0.1668	0.4302
4	Common palm civet	*Paradoxurus hermaphroditus*	0.2746	0.0773	0.1409	0.4397
5	Crab eating mongoose	*Herpestes urva*	0.5689	0.0938	0.3948	0.7599
6	Crested argus	*Rheinardia ocellata*	0.2745	0.1132	0.1042	0.5345
7	Eurasian wild pig	*Sus scrofa*	0.5618	0.0901	0.3899	0.7405
8	Leopard cat	*Prionailurus bengalensis*	0.4207	0.1289	0.2118	0.7253
9	Malayan porcupine	*Hystrix brachyura*	0.3596	0.1394	0.1404	0.6884
10	Northern pig-tailed macaque	*Macaca leonina*	0.3988	0.0843	0.2477	0.5774
11	Red muntjac	*Muntiacus vaginalis*	0.4170	0.0911	0.2561	0.6100
12	Owston's civet	*Chrotogale owstoni*	0.2491	0.1651	0.0137	0.6218
13	Pangolin	*Manis* spp.	0.2081	0.1471	0.0109	0.5366
14	Spotted linsang	*Prionodon pardicolor*	0.4193	0.1437	0.1842	0.7534
15	Yellow-throated marten	*Martes flavigula*	0.2788	0.1162	0.0983	0.5418

**Xe Sap / Palé (hunted / Vietnam)**						
**No.**	**Common name**	**Scientific name**	**Mean**	**SD**	**2.50%**	**97.50%**
1	Dark muntjac	*Muntiacus rooseveltorum / truongsonensis*	0.1416	0.0794	0.0445	0.3462
2	Asian black bear	*Ursus thibetanus*	0.1673	0.1485	0.0247	0.6216
3	Asiatic brush tailed porcupine	*Atherurus macrourus*	0.1224	0.0456	0.0498	0.2312
4	Common palm civet	*Paradoxurus hermaphroditus*	0.1040	0.0452	0.0326	0.2109
5	Crab eating mongoose	*Herpestes urva*	0.1224	0.0456	0.0498	0.2312
6	Crested argus	*Rheinardia ocellata*	0.0618	0.0437	0.0031	0.1577
7	Eurasian wild pig	*Sus scrofa*	0.1285	0.0460	0.0566	0.2387
8	Leopard cat	*Prionailurus bengalensis*	0.0795	0.0458	0.0118	0.1817
9	Malayan porcupine	*Hystrix brachyura*	0.0650	0.0466	0.0034	0.1683
10	Northern pig-tailed macaque	*Macaca leonina*	0.0742	0.0409	0.0116	0.1626

11	Red muntjac	*Muntiacus vaginalis*	0.1131	0.0454	0.0416	0.2218
12	Owston's civet	*Chrotogale owstoni*	0.1681	0.1475	0.0252	0.6236
13	Pangolin	*Manis* spp.	0.2707	0.1959	0.0694	0.8209
14	Spotted linsang	*Prionodon pardicolor*	0.2970	0.1727	0.0913	0.7413
15	Yellow-throated marten	*Martes flavigula*	0.1729	0.0979	0.0567	0.4341

**Deramakot FR (degraded / Malaysian Borneo)**						
**No.**	**Common name**	**Scientific name**	**Mean**	**SD**	**2.50%**	**97.50%**
1	Bornean yellow muntjac	*Muntiacus atherodes*	0.8603	0.0515	0.7468	0.9463
2	Sun bear	*Helarctos malayanus*	0.5132	0.0759	0.3671	0.6620
3	Long-tailed porcupine	*Trichys fasciculata*	0.2466	0.0669	0.1324	0.3917
4	Common palm civet	*Paradoxurus hermaphroditus*	0.1974	0.1348	0.0300	0.5467
5	Short-tailed mongoose	*Herpestes brachyurus*	0.4109	0.1537	0.1726	0.7711
6	Greater argus	*Argusianus argus*	0.6293	0.0833	0.4600	0.7846
7	Bearded pig	*Sus barbatus*	0.8856	0.0437	0.7871	0.9567
8	Leopard cat	*Prionailurus bengalensis*	0.1928	0.0736	0.0758	0.3605
9	Malay porcupine	*Hystrix brachyura*	0.4274	0.0688	0.2958	0.5653
10	Southern pig-tailed macaque	*Macaca nemestrina*	0.8740	0.0496	0.7655	0.9574
11	Southern red muntjac	*Muntiacus muntjak*	0.0509	0.0289	0.0111	0.1225
12	Banded civet	*Hemigalus derbyanus*	0.4211	0.1301	0.2170	0.7292
13	Sunda pangolin	*Manis javanica*	0.6585	0.2077	0.2556	0.9848
14	Banded linsang	*Prionodon linsang*	0.3930	0.2733	0.0351	0.9568
15	Yellow-throated marten	*Martes flavigula*	0.5345	0.2289	0.1483	0.9637

**Tangkulap FR (degraded / Malaysian Borneo)**						
**No.**	**Common name**	**Scientific name**	**Mean**	**SD**	**2.50%**	**97.50%**
1	Bornean yellow muntjac	*Muntiacus atherodes*	0.6252	0.0632	0.4981	0.7440

2	Sun bear	*Helarctos malayanus*	0.4612	0.0707	0.3255	0.6026
3	Long-tailed porcupine	*Trichys fasciculata*	0.3682	0.0785	0.2285	0.5337
4	Common palm civet	*Paradoxurus hermaphroditus*	0.4659	0.1830	0.1678	0.8589
5	Short-tailed mongoose	*Herpestes brachyurus*	0.1147	0.0752	0.0233	0.3053
6	Greater argus	*Argusianus argus*	0.2753	0.0636	0.1621	0.4089
7	Bearded pig	*Sus barbatus*	0.9495	0.0287	0.8818	0.9918
8	Leopard cat	*Prionailurus bengalensis*	0.3212	0.0853	0.1706	0.5043
9	Malay porcupine	*Hystrix brachyura*	0.5669	0.0650	0.4373	0.6934
10	Southern pig-tailed macaque	*Macaca nemestrina*	0.6357	0.0630	0.5102	0.7553
11	Southern red muntjac	*Muntiacus muntjak*	0.0120	0.0126	0.0002	0.0459
12	Banded civet	*Hemigalus derbyanus*	0.0983	0.0496	0.0280	0.2180
13	Sunda pangolin	*Manis javanica*	0.7895	0.1803	0.3786	0.9973
14	Banded linsang	*Prionodon linsang*	0.3116	0.2742	0.0145	0.9623
15	Yellow-throated marten	*Martes flavigula*	0.6276	0.2324	0.2001	0.9929

**Kuamut FR (degraded / Malaysian Borneo)**						
**No.**	**Common name**	**Scientific name**	**Mean**	**SD**	**2.50%**	**97.50%**
1	Bornean yellow muntjac	*Muntiacus atherodes*	0.6080	0.0741	0.4584	0.7476
2	Sun bear	*Helarctos malayanus*	0.4346	0.0807	0.2824	0.5974
3	Long-tailed porcupine	*Trichys fasciculata*	0.2760	0.0781	0.1425	0.4450
4	Common palm civet	*Paradoxurus hermaphroditus*	0.2005	0.1504	0.0227	0.5872
5	Short-tailed mongoose	*Herpestes brachyurus*	0.3924	0.1496	0.1592	0.7397
6	Greater argus	*Argusianus argus*	0.3509	0.0773	0.2111	0.5117
7	Bearded pig	*Sus barbatus*	0.9096	0.0415	0.8134	0.9729
8	Leopard cat	*Prionailurus bengalensis*	0.4277	0.0982	0.2497	0.6284
9	Malay porcupine	*Hystrix brachyura*	0.6822	0.0713	0.5361	0.8132
10	Southern pig-tailed macaque	*Macaca nemestrina*	0.8773	0.0502	0.7658	0.9606

11	Southern red muntjac	*Muntiacus muntjak*	0.4601	0.0791	0.3090	0.6160
12	Banded civet	*Hemigalus derbyanus*	0.4694	0.1311	0.2511	0.7673
13	Sunda pangolin	*Manis javanica*	0.6486	0.1830	0.2870	0.9607
14	Banded linsang	*Prionodon linsang*	0.5083	0.2447	0.0892	0.9486
15	Yellow-throated marten	*Martes flavigula*	0.7154	0.1792	0.3247	0.9789

Supplementary Table 4: Posterior summary statistics showing covariate effect sizes for estimated occupancy for the 15 species and sister species pairs.

Village density: hunted sites (Bach Ma NP, Hue and Quang Nam SNRs, Xe Sap / Palé)							
**No.**	**Common name**	**Scientific name**	**Mean**	**2.50%**	**25%**	**75%**	**97.50%**
1	Dark muntjac	*Muntiacus rooseveltorum / truongsonensis*	-0.7700	-1.7422	-1.0271	-0.4670	-0.0329
2	Asian black bear	*Ursus thibetanus*	-0.5329	-1.7785	-0.8537	-0.1608	0.4830
3	Asiatic brush tailed porcupine	*Atherurus macrourus*	-0.3307	-0.7612	-0.4720	-0.1867	0.0817
4	Common palm civet	*Paradoxurus hermaphroditus*	-0.0776	-0.5520	-0.2378	0.0790	0.3954
5	Crab eating mongoose	*Herpestes urva*	-0.1702	-0.6582	-0.3302	-0.0089	0.3001
6	Crested argus	*Rheinardia ocellata*	0.0518	-0.6639	-0.1961	0.2919	0.8042
7	Eurasian wild pig	*Sus scrofa*	-0.9546	-1.5368	-1.1342	-0.7612	-0.4492
8	Leopard cat	*Prionailurus bengalensis*	-0.4225	-1.1269	-0.6356	-0.2029	0.2168
9	Malayan porcupine	*Hystrix brachyura*	-0.6407	-1.5651	-0.9019	-0.3491	0.1266
10	Northern pig-tailed macaque	*Macaca leonina*	0.4240	-0.0287	0.2602	0.5812	0.9111
11	Red muntjac	*Muntiacus vaginalis*	-0.8116	-1.5570	-1.0338	-0.5683	-0.1684
12	Owston's civet	*Chrotogale owstoni*	-0.4988	-1.7446	-0.8225	-0.1338	0.5229
13	Pangolin	*Manis* spp.	-0.7224	-2.0208	-1.0340	-0.3488	0.2459
14	Spotted linsang	*Prionodon pardicolor*	0.0045	-0.6613	-0.2153	0.2208	0.6840
15	Yellow-throated marten	*Martes flavigula*	-0.2732	-0.9354	-0.4786	-0.0577	0.3431

Canopy closure: hunted sites (Bach Ma NP, Hue and Quang Nam SNRs, Xe Sap / Palé)							
**No.**	**Common name**	**Scientific name**	**Mean**	**2.50%**	**25%**	**75%**	**97.50%**
1	Dark muntjac	*Muntiacus rooseveltorum / truongsonensis*	0.3405	-0.1843	0.1300	0.5194	1.0321
2	Asian black bear	*Ursus thibetanus*	0.0491	-0.6372	-0.1571	0.2545	0.7373
3	Asiatic brush tailed porcupine	*Atherurus macrourus*	0.0265	-0.3584	-0.0997	0.1525	0.4164
4	Common palm civet	*Paradoxurus hermaphroditus*	-0.1062	-0.5523	-0.2458	0.0406	0.3062
5	Crab eating mongoose	*Herpestes urva*	-0.0854	-0.5044	-0.2192	0.0538	0.3128

6	Crested argus	*Rheinardia ocellata*	0.2503	-0.2836	0.0514	0.4261	0.9020
7	Eurasian wild pig	*Sus scrofa*	0.0394	-0.3515	-0.0889	0.1669	0.4303
8	Leopard cat	*Prionailurus bengalensis*	0.0523	-0.4595	-0.1119	0.2168	0.5591
9	Malayan porcupine	*Hystrix brachyura*	-0.1316	-0.7607	-0.3063	0.0591	0.3991
10	Northern pig-tailed macaque	*Macaca leonina*	0.1734	-0.2456	0.0261	0.3149	0.6203
11	Red muntjac	*Muntiacus vaginalis*	0.0933	-0.3812	-0.0654	0.2474	0.5812
12	Owston's civet	*Chrotogale owstoni*	0.0459	-0.6409	-0.1628	0.2516	0.7407
13	Pangolin	*Manis* spp.	0.1075	-0.5200	-0.0965	0.3029	0.7895
14	Spotted linsang	*Prionodon pardicolor*	0.0171	-0.5622	-0.1570	0.1939	0.5852
15	Yellow-throated marten	*Martes flavigula*	-0.0407	-0.5437	-0.2002	0.1225	0.4453

**Canopy closure: degraded sites (Deramakot FR, Tangkulap FR, Kuamut FR)**							
**No.**	**Common name**	**Scientific name**	**Mean**	**2.50%**	**25%**	**75%**	**97.50%**
1	Bornean yellow muntjac	*Muntiacus atherodes*	0.4874	0.1464	0.3657	0.6053	0.8479
2	Sun bear	*Helarctos malayanus*	0.3351	-0.0249	0.2069	0.4596	0.7158
3	Long-tailed porcupine	*Trichys fasciculata*	0.7297	0.2284	0.5359	0.9070	1.3176
4	Common palm civet	*Paradoxurus hermaphroditus*	-2.0041	-4.5704	-2.5447	-1.2403	-0.5766
5	Short-tailed mongoose	*Herpestes brachyurus*	-0.8158	-1.6409	-1.0217	-0.5661	-0.1955
6	Great argus	*Argusianus argus*	1.4879	0.8511	1.2365	1.7166	2.2639
7	Bearded pig	*Sus barbatus*	-0.1800	-1.1351	-0.3812	0.0844	0.4494
8	Leopard cat	*Prionailurus bengalensis*	-2.1185	-3.1255	-2.3890	-1.8044	-1.3541
9	Malay porcupine	*Hystrix brachyura*	0.0174	-0.3247	-0.0950	0.1313	0.3494
10	Southern pig-tailed macaque	*Macaca nemestrina*	0.1520	-0.2629	0.0225	0.2897	0.5321
11	Southern red muntjac	*Muntiacus muntjak*	-0.4862	-0.9989	-0.6523	-0.3151	-0.0082
12	Banded civet	*Hemigalus derbyanus*	0.9186	0.1944	0.6204	1.1662	1.8910
13	Sunda pangolin	*Manis javanica*	0.3691	-1.1169	-0.0277	0.8035	1.6691
14	Banded linsang	*Prionodon linsang*	1.2202	-0.5758	0.5548	1.8095	3.4785
15	Yellow-throated marten	*Martes flavigula*	-0.2650	-2.2044	-0.7708	0.3101	1.2732

Village density: degraded sites (Deramakot FR, Tangkulap FR, Kuamut FR)							
**No.**	**Common name**	**Scientific name**	**Mean**	**2.50%**	**25%**	**75%**	**97.50%**
1	Bornean yellow muntjac	*Muntiacus atherodes*	0.2677	-0.0856	0.1403	0.3891	0.6564
2	Sun bear	*Helarctos malayanus*	0.1995	-0.1217	0.0852	0.3099	0.5402
3	Long-tailed porcupine	*Trichys fasciculata*	-0.0820	-0.4507	-0.1997	0.0402	0.2652
4	Common palm civet	*Paradoxurus hermaphroditus*	0.0273	-0.4861	-0.1341	0.1893	0.5394
5	Short-tailed mongoose	*Herpestes brachyurus*	0.1260	-0.3214	-0.0242	0.2704	0.5964
6	Great argus	*Argusianus argus*	-0.0207	-0.3725	-0.1359	0.0966	0.3148
7	Bearded pig	*Sus barbatus*	0.0543	-0.3681	-0.0872	0.1914	0.4893
8	Leopard cat	*Prionailurus bengalensis*	-0.1728	-0.6317	-0.3088	-0.0235	0.2278
9	Malay porcupine	*Hystrix brachyura*	-0.0088	-0.3249	-0.1135	0.0969	0.3022
10	Southern pig-tailed macaque	*Macaca nemestrina*	-0.0562	-0.4368	-0.1781	0.0685	0.3087
11	Southern red muntjac	*Muntiacus muntjak*	0.1388	-0.2950	-0.0104	0.2833	0.5972
12	Banded civet	*Hemigalus derbyanus*	0.1547	-0.2388	0.0164	0.2884	0.5787
13	Sunda pangolin	*Manis javanica*	0.0253	-0.5304	-0.1525	0.2018	0.5895
14	Banded linsang	*Prionodon linsang*	-0.0021	-0.6160	-0.1861	0.1871	0.5832
15	Yellow-throated marten	*Martes flavigula*	0.0504	-0.4978	-0.1289	0.2240	0.6226

Supplementary Table 5: Details of camera-trap surveys conducted in hunted (Vietnam / Laos) and degraded sites (Malaysian Borneo), with study site name, survey dates, number of camera trap stations per site, and camera trap effort per site.

Landscape	Site	Survey dates	No. camera trap stations	No. camera-trap nights
Hunted sites	Bach Ma NP	Nov. 2014 - Jan. 2015	53	6,805
	Hue and Quang Nam SNRs	Aug. - Dec. 2015	44	5,270
	Xe Sap / Palé	July - Dec. 2016	39	4,967
		**Total**	**136**	**17,042**
Degraded sites	Deramakot	Oct. 2014 - Jan. 2015	63	8,909
	Northern Kuamut	Mar. - Jul. 2016	53	6,865
	Tangulap-Sungai Talibu	Aug. - Nov. 2015	64	8,396
		**Total**	**180**	**24,170**

References for Supplemental Information material

1. Francis, C. (2019). *Field guide to the mammals of South-east Asia*. Bloomsbury Publishing.

2. Phillipps, Q. (2011). *Phillipps' field guide to the birds of Borneo*. Oxford, England: John Beaufoy.

3. Phillipps, Q. (2016). *Phillipps' field guide to the mammals of Borneo and their ecology: Sabah, Sarawak, Brunei, and Kalimantan (Vol. 105)*. Princeton University Press.
