# Supplementary material for: Habitat degradation and indiscriminate hunting differentially impact faunal communities in the Southeast Asian tropical biodiversity hotspot
Source: Commun Biol. 2019 Oct 30;2:396. doi: 10.1038/s42003-019-0640-y (PMC6821809; doi:10.1038/s42003-019-0640-y)
Supplement: Supplementary file 2 — Reporting Summary [file 42003_2019_640_MOESM2_ESM.pdf]

nature research

Corresponding author(s): Andrew Tilker and Jesse F. Abrams

Last updated by author(s): Aug 28, 2019

Reporting Summary

Nature Research wishes to improve the reproducibility of the work that we publish. This form provides structure for consistency and transparency in reporting. For further information on Nature Research policies, see Authors & Referees and the Editorial Policy Checklist.

Statistics

For all statistical analyses, confirm that the following items are present in the figure legend, table legend, main text, or Methods section.

n/a	Confirmed	
☐	☒	The exact sample size (*n*) for each experimental group/condition, given as a discrete number and unit of measurement
☒	☐	A statement on whether measurements were taken from distinct samples or whether the same sample was measured repeatedly
☒	☐	The statistical test(s) used AND whether they are one- or two-sided<br>*Only common tests should be described solely by name; describe more complex techniques in the Methods section.*
☐	☒	A description of all covariates tested
☐	☒	A description of any assumptions or corrections, such as tests of normality and adjustment for multiple comparisons
☐	☒	A full description of the statistical parameters including central tendency (e.g. means) or other basic estimates (e.g. regression coefficient) AND variation (e.g. standard deviation) or associated estimates of uncertainty (e.g. confidence intervals)
☒	☐	For null hypothesis testing, the test statistic (e.g. *F*, *t*, *r*) with confidence intervals, effect sizes, degrees of freedom and *P* value noted<br>*Give P values as exact values whenever suitable.*
☐	☒	For Bayesian analysis, information on the choice of priors and Markov chain Monte Carlo settings
☐	☒	For hierarchical and complex designs, identification of the appropriate level for tests and full reporting of outcomes
☐	☒	Estimates of effect sizes (e.g. Cohen's *d*, Pearson's *r*), indicating how they were calculated

Our web collection on statistics for biologists contains articles on many of the points above.

Software and code

Policy information about availability of computer code

Data collection	No software was used in data collection.
Data analysis	All data analyses were performed in R v. 3.4.0.

For manuscripts utilizing custom algorithms or software that are central to the research but not yet described in published literature, software must be made available to editors/reviewers. We strongly encourage code deposition in a community repository (e.g. GitHub). See the Nature Research guidelines for submitting code & software for further information.

Data

Policy information about availability of data

All manuscripts must include a data availability statement. This statement should provide the following information, where applicable:

- Accession codes, unique identifiers, or web links for publicly available datasets
- A list of figures that have associated raw data
- A description of any restrictions on data availability

The data used in this study is not publicly archived because it contains information on the locations of Red Listed as well as hunted and traded species. However, all data in support of the findings of this study are available from the corresponding author by reasonable request.

Field-specific reporting

Please select the one below that is the best fit for your research. If you are not sure, read the appropriate sections before making your selection.

☐ Life sciences ☐ Behavioural & social sciences ☒ Ecological, evolutionary & environmental sciences

Ecological, evolutionary & environmental sciences study design

All studies must disclose on these points even when the disclosure is negative.

Study description	We assess how different drivers of defaunation impact tropical terrestrial mammal and bird communities in the Southeast Asian biodiversity hotpot. We conducted systematic landscape-scale camera trapping in two landscapes characterized by different defaunation drivers In Malaysian Borneo, we sampled three individual study sites characterized by moderate levels of habitat degradation through logging. Hunting in these sites is minimal. In the central Annamites (Vietnam and Laos) we sampled five study sites that have experienced severe past and current hunting levels of pressure through the setting of indiscriminate wire snares. The forest in these sites is structurally intact, with minimal degradation. We assess defaunation at three heirarchical levels: (1) species functional extinctions, (2) species' occupancy for species that occurred in both the Annamites and Malaysian Borneo, and (3) predictors of species occurrence.
Research sample	Ground-dwelling mammal and bird communities in the Annamites (Vietnam / Laos) and Malaysian Borneo.
Sampling strategy	No predetermined sample size. Detections of terrestrial mammal and bird species were sufficient for model convergence in the multi-species occupancy analyses.
Data collection	We sampled mammalian and ground-dwelling bird communities by setting camera-traps. We used a systematic, landscape-scale approach, with camera-trap stations spaced approximately 2.5 km apart (Annamites: $\bar{x}$ = 2.47 ± 0.233 km; Malaysian Borneo: $\bar{x}$ = 2.46 ± 0.220 km) across the study sites. To increase detection probabilities, we set two white-flash camera-traps (Reconyx® Hyperfire Professional PC850; Reconyx, Holmen, USA) at each sampling station. Cameras were set in different directions. All cameras were placed 20 – 40 cm above the ground and were operational 24 hours per day. Camera-trap data was later retrieved and processed using the R package camtrapR.
Timing and spatial scale	Systematic camera trapping in the Annamites was conducted between November 2014 and December 2016. Systematic camera trapping in Malaysian Borneo was conducted between October 2014 and July 2016. Cameras were left in the field for a minimum of 60 days.
Data exclusions	No data excluded from the analysis.
Reproducibility	Camera-trap setup procedures were standardized prior to the study to ensure reproducibility.
Randomization	We did not allocate samples into groups in this study.
Blinding	We acquired data from camera-trapping. No blinding was required.

Did the study involve field work? ☒ Yes ☐ No

Field work, collection and transport

Field conditions	Study sites in both Vietnam / Laos and Malaysian Borneo were characterized by close-canopy wet evergreen tropical rainforest.
Location	(1) Central Annamites landscape, Vietnam and Lao PDR. Five study sites: Bach Ma National Park, Hue Saola Nature Reserve, Quang Nam Saola Nature Reserve, eastern section of Xe Sap National Protected Area, and the Palé area. Approximate latitude and longitude coordinates: 16° 4'28.59"N, 107°34'12.25"E. (2) Sabah, Malaysia, Borneo. Three study sites: Deramakot Forest Reserve, Tangkulap Forest Reserve, and Kuamut Forest Reserve. pproximate latitude and longitude coordinates: 5°19'23.57"N, 117°17'58.80".
Access and import/export	All fieldwork was conducted under the WWF's Carbon & Biodiversity (CarBi) project. Permission to conduct fieldwork in Bach Ma NP and the Hue Saola Nature Reserve (both in Thua Thien Hue province, Vietnam) was provided by permit 2170/QD-UBND, 24/10/2014, granted by the Thua Thien Hue Provincial People's Committee. Permission to conduct fieldwork in the Quang Nam Saola Nature Reserve (Quang Nam Province) was provided by permits 725/QD-UBND, 01/04/2011 granted by the Quang Nam Provincial People's Committee. Permission in Lao PDR was granted through an Memorandum of Understanding (MOU) between WWF-Laos and the government of Lao PDR.
Disturbance	None. We used noninvasive camera-trapping to record wildlife photographs.

Reporting for specific materials, systems and methods

We require information from authors about some types of materials, experimental systems and methods used in many studies. Here, indicate whether each material, system or method listed is relevant to your study. If you are not sure if a list item applies to your research, read the appropriate section before selecting a response.

Materials & experimental systems

n/a	Involved in the study
☒	☐ Antibodies
☒	☐ Eukaryotic cell lines
☒	☐ Palaeontology
☐	☒ Animals and other organisms
☒	☐ Human research participants
☒	☐ Clinical data

Methods

n/a	Involved in the study
☒	☐ ChIP-seq
☒	☐ Flow cytometry
☒	☐ MRI-based neuroimaging

Animals and other organisms

Policy information about studies involving animals; ARRIVE guidelines recommended for reporting animal research

Laboratory animals	N/A
Wild animals	We used non-invasive camera-trapping to record photographs of ground-dwelling mammal and bird species in our study sites. The animals were not captured or handled in any way.
Field-collected samples	N/A
Ethics oversight	No ethical oversight required.

Note that full information on the approval of the study protocol must also be provided in the manuscript.
